# Cost‐Effective Na_4_Fe_3_(PO_4_)_2_P_2_O_7_ Cathode Materials for Sodium‐Ion Batteries in Large‐Scale Energy Storage Applications

**DOI:** 10.1002/smsc.70316

**Published:** 2026-06-29

**Authors:** Qinghua Shen, Yonghong Qin, Boyi Pang, Ruilin Mai, Jin Song, Huanxin Li, Chaopeng Fu

**Affiliations:** ^1^ School of Materials Science and Engineering Shanghai Jiao Tong University Shanghai China; ^2^ Advanced Propulsion Lab (APL) University College London London UK; ^3^ Electrochemical Innovation Lab (EIL) University College London London UK

## Abstract

The rising interest in sodium‐ion batteries (SIBs) has led to significant research on cathode materials, given their essential function in SIBs. Among various cathode materials, Na_4_Fe_3_(PO_4_)_2_P_2_O_7_ (NFPP) stands out with its NASICON structure, offering high structural stability, rapid ion transport, and high operating potential. With its ultralong cycle life, cost‐effectiveness, high safety, and superb temperature adaptability, NFPP is an attractive choice for large‐scale energy storage. Despite these advantages, the widespread application of NFPP is limited by low electronic conductivity and impurity issues. In this review, we discussed the crystal structure, properties, and diverse synthetic approaches of NFPP. Additionally, various modification strategies are also looked into including impurity adjustment, doping, and surface modification. The full cells of NFPP were further analyzed, underscoring its potential for commercialization. In conclusion, we outline our opinions on the obstacles and future research priorities for NFPP. This review aims to assess the potential of NFPP for large‐scale energy storage applications, sparking greater interest and progress in NFPP cathode materials.

## Graphical abstract

Na_4_Fe_3_(PO_4_)_2_P_2_O_7_ (NFPP) cathodes for sodium‐ion batteries are systematically reviewed, covering crystal structure, synthesis strategies, and electrochemical properties. Key challenges, including low electronic conductivity and impurity control, are critically analyzed alongside recent modification approaches. Insights into structure–property relationships provide guidance for rational design and support the development of scalable, safe, and durable energy storage systems.

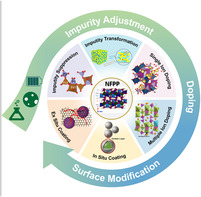

## 1 Introduction

As part of the global energy transformation plan, wind and solar power generation are advancing rapidly, accelerating to replace coal‐fired power generation [1, 2]. Nonetheless, grids cannot fully take advantage of wind and solar power due to their inconsistency and instability, resulting in significant electricity waste [3, 4]. To address this challenge, lithium‐ion batteries (LIBs) are currently considered the leading energy storage technology because of their high energy density and cycle stability [5, 6]. Although widely used, LIBs face challenges in large‐scale energy storage due to limited lithium resources and cost concerns [7]. To overcome these barriers, sodium‐ion batteries (SIBs), a more affordable and resource‐abundant alternative, are gaining increasing attention [4, 8]. The advancement of SIB technology can be expedited by directly applying strategies designed for LIBs, as they both operate on the ’rocking chair’ mechanism [9, 10]. Nonetheless, enhancing the energy density and cycling stability of SIBs remains crucial for meeting the demands of practical applications.

SIBs require Na^+^ ions to be reversibly embedded and de‐embedded between cathode and anode electrodes to achieve energy conversion during the charging and discharging [11]. Consequently, the electrochemical performances of SIBs are significantly affected by the properties of the electrode materials, particularly cathode materials [12]. Different cathode materials have been designed, including layered oxides, Prussian blue analogs, and polyanion‐type compounds [13, 14, 15]. Although layered oxides exhibit exceptional energy density, their high sensitivity to humidity and low redox potential limit their suitability for extensive energy storage applications [16, 17]. Besides, Prussian blue analogs garnered attention for being cost‐effective, while their stability can be compromised by ligand water and lattice defects [18, 19]. In contrast, polyanion‐type compounds are increasingly studied and utilized due to their high safety and superior electrochemical properties [20, 21, 22]. The outstanding voltage platform, cycling performance, and thermal stability of polyanion compounds also make them promising candidates for energy storage despite their low conductivity issues [23, 24].

Among various polyanion types, iron‐based cathode materials, particularly Na_4_Fe_3_(PO_4_)_2_P_2_O_7_ (NFPP), distinguish them from other polyanionic options because of their affordability and abundant resources [25]. In recent years, NFPP cathode material has gained prominence as a leading candidate for commercial energy storage technology in SIBs. With its unique structure, NFPP offers a voltage of around 3.2 V and a theoretical capacity of 129 mAh g^−1^, demonstrating exceptional stability beyond 10 000 cycles [26]. Although NFPP is still a developing material, significant advancements toward its commercialization have been achieved [27, 28]. Both academic and industrial communities continue to monitor its long‐term performance closely, despite the rapid pace of commercialization [29]. Despite its promising properties, NFPP still faces several critical challenges that limit its practical application. First, its intrinsically low electronic conductivity leads to poor rate capability, requiring additional modification strategies. Second, impurity phase formation during synthesis is difficult to control and can significantly affect electrochemical performance. Third, achieving scalable and cost‐effective production while maintaining phase purity and structural uniformity remains a major engineering challenge. Addressing these issues is essential for the practical deployment of NFPP‐based cathodes and constitutes the main focus of current research. The lack of comprehensive information has impeded a deeper understanding of NFPP. To bridge this gap and guide future material design, it is crucial to provide a thorough summary and analysis of the research progress on NFPP.

This review reveals the fundamental details of NFPP by analyzing its crystal structure, material properties, and synthesis methods. Strategies for enhancing its electrochemical performance, including impurity adjustment, doping, and surface modification, are also discussed. The full cell and commercialization of NFPP were also examined, underscoring its potential for energy storage applications. Finally, the challenges and future research emphasis for NFPP are proposed. This review aims to provide readers with a thorough understanding of NFPP and inspire further research efforts.

## 2 Crystal Structure and Properties of Na_4_Fe_3_(PO_4_)_2_(P_2_O_7_)

**FIGURE 1:**
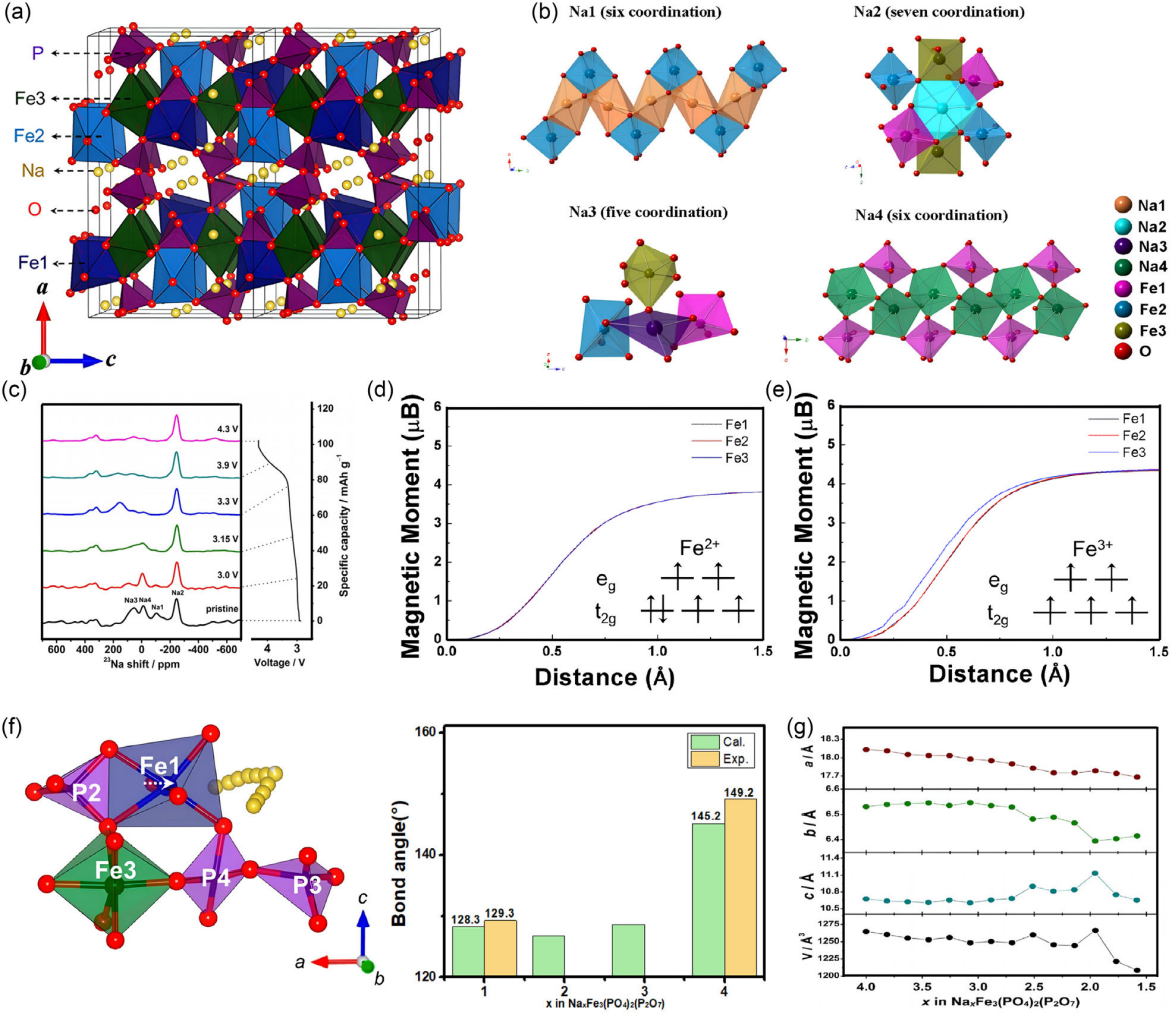
(a) Schematic illustration of Na_4_Fe_3_(PO_4_)_2_(P_2_O_7_) [30]. Reproduced with permission [31]. Copyright 2012, American Chemical Society. (b) Schematic depiction of coordination number and local structure of Na^+^ in NFPP [31]. (c) 23Na MAS NMR spectra of NFPP electrodes at various charge states [31]. Reproduced with permission [32]. Copyright 2019, Elsevier. In Na_*x*_Fe_3_(PO_4_)_2_(P_2_O_7_), (d) *x* = 4 and (e) *x* = 1 display integrated spin as a function of integration radius around Fe [32]. Reproduced with permission [33]. Copyright 2013, American Chemical Society. (f) Schematic of the local structure around Fe1 and the P(3)−O−P(4) bond angle comparison diagram as a function of Na content x in Na_*x*_Fe_3_(PO_4_)_2_(P_2_O_7_) from ND analysis [32]. Reproduced with permission [33]. Copyright 2013, American Chemical Society. (g) The lattice parameters of NFPP as a function of sodium concentration x, estimated from the electrochemical charging current during the initial charging process [31]. Reproduced with permission [32]. Copyright 2019, Elsevier.

In 2012, the crystal structure (Figure 1a) and properties of Na_4_Fe_3_(PO_4_)_2_(P_2_O_7_) were studied by Kim et al. [30]. It was discovered that NFPP possesses an orthorhombic crystal system with a Pn2_1_a space group and structural features similar to NASICON‐type crystals. Rietveld refinement provided further information about the cell parameters and volume characteristics of NFPP (*a* = 18.07517 Å, *b* = 6.53238 Å, *c* = 10.64760 Å, and *V* = 1257.204 Å^3^). In this structure, an infinite layer structure [Fe_3_P_2_O_13_]∞ is formed within the bc plane, composed of FeO_6_ octahedra and (PO_4_)^3−^ groups, which is interconnected by P_2_O_7_ groups along the *a*‐axis, resulting in an open and free three‐dimensional (3D) framework [32]. The [PO_4_] and [P_2_O_7_] units within the open frame make NFPP unique by allowing Na^+^ ions to diffuse through a 3D pathway, in contrast to many polyanion compounds that restrict diffusion to one or two‐dimensional [33, 34, 35]. Nonetheless, these insulating units simultaneously hinder electron transport, negatively impacting the electrochemical performance of NFPP.

Four Na sites (Na1, Na2, Na3, and Na4) are located around Fe sites in NFPP (Figure 1b), indicating the electrochemical capacity of NFPP due to their distinct coordination environments and exchangeability [31]. As illustrated by the ex situ solid‐state nuclear magnetic resonance (NMR) spectra (Figure 1c), Na^+^ ions in the NFPP structure experience different coordination settings: Na1 and Na4 are coordinated with six atoms, Na2 with seven, and Na3 with five. Notably, Na^+^ ions that have lower coordination numbers are more easily extracted than those with higher ones. Specifically, the extraction sequence is based on the increase in coordination number: The five‐coordinated Na3 is extracted first due to its higher chemical potential, followed by the six‐coordinated Na1 and Na4, whereas the seven‐coordinated Na2 primarily stabilizes the crystal structure and seldom engages in the reaction. It is worth noting that, unlike the extraction order of Na^+^ ions by the sol–gel method, Kim et al. demonstrated a distinct extraction order using the solid‐state method combined with theoretical calculations [32]. Their study indicates that the Na^+^ ions from Na3 (five‐coordination) and Na1 (six‐coordination) were initially extracted. Subsequently, some Na^+^ ions from Na4 (six‐coordination) and Na2 (seven‐coordination) sites are removed, ultimately leaving half of the Na4 and Na2 sites in the NaFe_3_(PO_4_)_2_(P_2_O_7_) structure. To ensure the consistency and accuracy of these findings, additional experimental validation and theoretical analysis may be necessary to explain the differences.

The oxidation state of the Fe sites in the NFPP structure changes simultaneously with Na extraction. As illustrated in Figure 1d,e, the oxidation state of the transition metal is indicated by its magnetic moment around it. Due to electrostatic interactions between Fe and their relative position to Na sites, the Fe1 site oxidizes first to Fe^3+^, followed by the Fe2 site, with the Fe3 site oxidizing at the last step [32]. First‐principles calculations revealed that P_2_O_7_ undergoes significant distortion as the *x*‐value changes from 2 to 1 (Figure 1f). Simultaneously, the FeO6 polyhedra connecting the Fe1 and Fe3 sites change from edge‐sharing to corner‐sharing, primarily because of electrostatic repulsion among the Fe3, causing an unusual decrease in the *c*‐lattice parameter depicted in Figure 1g. The diffusion tunneling of Na narrows due to local structural deformations, particularly the movement of Fe in Fe1 and P_2_O_7_ dimers, leading to decreased Na de/embedding kinetics in certain regions and increased cell polarization. These findings shed light on the structure basis and kinetic mechanism of NFPP, which exhibit unique electrochemical performance in SIBs and provide valuable theoretical insights for optimizing cathode material design.

To further understand the discrepancies in Na^+^ extraction sequences reported for NFPP synthesized via different routes (e.g., solid‐state vs. sol–gel methods), it is important to consider both kinetic and thermodynamic factors. From a kinetic perspective, synthesis‐dependent variations in particle size, morphology, and crystallinity can significantly influence Na^+^ diffusion pathways and activation barriers. For instance, sol–gel‐derived NFPP typically exhibits smaller particle sizes and shorter diffusion lengths, which may favor kinetically accessible Na^+^ extraction pathways that differ from those in bulkier solid‐state materials. In contrast, thermodynamic contributions arise from subtle differences in crystal chemistry, including lattice distortions, defect concentrations (e.g., Na vacancies or Fe nonstoichiometry), and trace impurities introduced during synthesis. These factors can modify the relative stability of intermediate phases during desodiation, thereby altering the preferred Na^+^ extraction sequence. In particular, defect‐induced local structural heterogeneity and cation disorder may redistribute the Na^+^ site energies, leading to deviations from the ideal extraction order predicted for a perfectly ordered lattice. Therefore, the observed differences in Na^+^ extraction behavior are likely governed by a coupling of kinetic pathway dependence and synthesis‐induced thermodynamic perturbations, rather than a single dominant mechanism.

**FIGURE 2:**
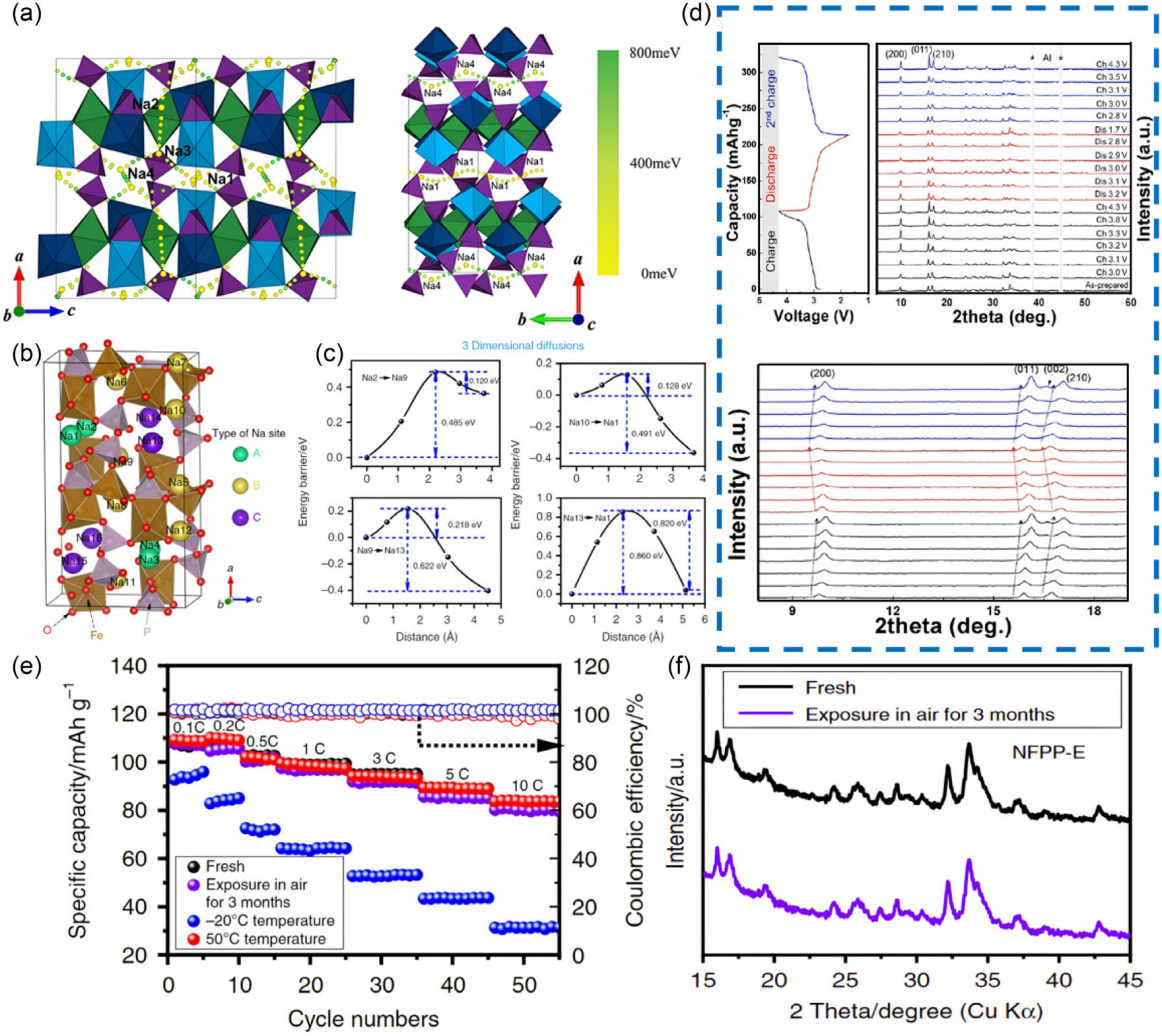
(a) Three‐dimensional diffusion path and migration barrier of Na^+^ ions in Na_4_Fe_3_(PO_4_)_2_(P_2_O_7_) structure [37]. Reproduced with permission [38]. Copyright 2024, Wiley. (b) Illustration of NFPP's crystal structure featuring three different types of Na^+^ ions [36]. Reproduced with permission [37]. Copyright 2019, Springer Nature. (c) Migration energy barrier between different Na^+^ ion groups corresponding to a three‐dimensional diffusion pathway [36]. Reproduced with permission [37]. Copyright 2019, Springer Nature. (d) Ex situ XRD patterns of NFPP during the charge/discharge process and amplified XRD patterns in the 2θ range 8°−19° [32]. Reproduced with permission [33]. Copyright 2013, American Chemical Society. (e) C‐rate capability of NFPP‐E electrodes in various states: fresh, exposed to air for 3 months, and fresh at both −20°C and 50°C [36]. Reproduced with permission [37]. Copyright 2019, Springer Nature. (f) XRD comparison of NFPP‐E powder in its fresh state and after 3 months of air exposure [36]. Reproduced with permission [37]. Copyright 2019, Springer Nature.

The localized migration energy barriers within the crystal structure of NFPP have been investigated using bond valence sum (BVS) and density functional theory (DFT) calculations, revealing the fundamental mechanism behind its exceptional rate performance [36]. It has been demonstrated that the crystal structure of NFPP offers a multidimensional pathway for the diffusion of Na^+^ ions diffusion (Figure 2a). Surprisingly, the *a*‐axis direction presents an insignificant diffusion energy barrier for Na^+^ ions, measuring only 0.02 eV. The calculated 3D diffusion paths for various types of Na^+^ ions in NFPP indicate that all Na^+^ ions exhibit diffusion energy barriers below 0.9 eV (Figure 2b,c). This evidence highlights mechanisms for the remarkable high‐rate performance of the modified materials. Furthermore, the study discovered that the overall conductivity of NFPP material is comparable to the well‐known NASICON‐type Na_3_V_2_(PO_4_)_3_ cathode material, as demonstrated through ionic conductivity tests, providing strong support for the fast ion transport properties of NFPP [38, 39].

The excellent structural stability of NFPP plays a significant role in its long‐term operational lifespan, resulting in an ultralong cycle life. The consistent patterns observed in ex situ XRD patterns (Figure 2d) indicate a topotactic Na removal, demonstrating that the structure of NFPP can be fully reversed. Despite the significant deformation of pyrophosphate (P_2_O_7_) polyhedra, the volume change of NFPP is constrained to under 4%, marking one of the lowest volume changes for the cathode materials to date [32]. This minimal volume change can be attributed to the openness and rigidity of the hybrid polyanion crystal structure. Specifically, the P_2_O_7_ dimer within the crystal structure can rotate and twist, effectively accommodating structural changes. Phase changes are associated with volume expansion and contraction, which can induce mechanical stresses leading to material structure degradation. Notably, the sodiation/desodiation redox process of NFPP is an imperfect single‐phase reaction, which is rare among polyanionic cathode materials. In contrast, other polyanion compounds based on iron redox reactions, such as NaFePO_4_ and Na_2_FeP_2_O_7_, are more likely to undergo biphasic reactions [40, 41]. These distinctive features provide a robust foundation for the long‐term stability of NFPP and highlight its great potential in energy storage systems.

The excellent temperature adaptability and thermal stability of NFPP distinguish it in the energy storage field, especially when compared with cobalt and nickel‐based materials. Nano‐Na_4_Fe_3_(PO_4_)_2_(P_2_O_7_) plates (NFPP‐E) were successfully synthesized via the sol–gel method, and their cycling stability was tested at extreme high or low temperatures of 50°C and −20°C (Figure 2e) [36]. Under these conditions, their capacity retention of NFPP‐E was still 91.4% and 92.1%, respectively, demonstrating that the crystal structures almost remain unchanged in both cases. In the low‐temperature test at −20°C, NFPP‐E maintained a specific capacity of 95.0 and 84.7 mAh g^−1^ at 0.1C and 0.2C. Additionally, the thermal stability of NFPP was thoroughly investigated, revealing the decomposition temperature of NFPP is approximately 530°C, higher compared to the conventional operating temperatures [32]. This substantially reduces the risk of thermal runaway, providing NFPP with distinct safety advantages. This inherent safety feature, combined with its applicability across all climate conditions, establishes a robust foundation for the commercialization of NFPP.

The air stability of NFPP cathode material is another significant advantage for its commercial application. As demonstrated in Figure 2f, the crystal structure and electrochemical properties of NFPP remained stable even after 3 months of air exposure, confirming its excellent air stability [36]. This advantage can further reduce overall costs by simplifying the battery manufacturing process, minimizing storage requirements, and enhancing the material's practicality. Additionally, recent studies have further explored the air stability of NFPP, revealing that it maintains stability when exposed to room‐temperature air for an hour. However, its electrochemical performance significantly deteriorates when subjected to temperatures of 80°C, 160°C, and 240°C for the same duration. Notably, moisture can cause ion leaching, leading to impurities that degrade battery efficiency [42]. To preserve the integrity and performance of NFPP, researchers advise limiting air exposure before use and storing it in low humidity or inert gas environments.

## 3 Methods for the Synthesis of Na_4_Fe_3_(PO_4_)_2_(P_2_O_7_)

The synthesis process is crucial in determining the structure and morphology of NFPP material, which further affects its electrochemical performance. Several reliable synthesis methods have been established, including solid‐state, spray‐drying, sol–gel, electrospinning, solution combustion, and template methods. Each of these methods offers distinct features and provides a variety of approaches for preparing NFPP materials.

### 3.1 Solid‐State Method

**FIGURE 3:**
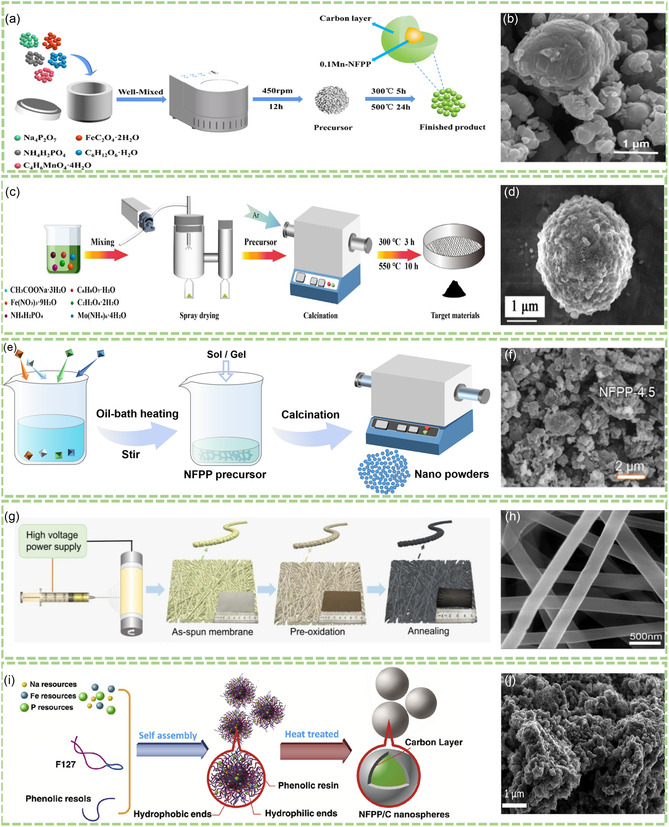
Preparation schematic of (a) the solid‐state method [43]. Reproduced with permission [45]. Copyright 2023, American Chemical Society. (c) The spray‐drying method [44]. Reproduced with permission [46]. Copyright 2024, MDPI. (e) The sol–gel method. (g) The electrospinning method [45]. Reproduced with permission [47]. Copyright 2023, Elsevier. (i) Template method [46]. Reproduced with permission [48]. Copyright 2019, Elsevier. The corresponding morphology of (b) NFMPP [47]. Reproduced with permission [49]. Copyright 2023, Elsevier. (d) NFPP@rGO [48]. Reproduced with permission [49]. Copyright 2019, Elsevier. (f) NFPP‐4.5 [49]. Reproduced with permission [51]. Copyright 2024, American Chemical Society. (h) NFPP@C [45]. Reproduced with permission [47]. Copyright 2023, Elsevier. (j) NFPP@HC [46]. Reproduced with permission [48]. Copyright 2019, Elsevier.

The solid‐state method involves mixing and grinding solid raw materials, followed by high‐temperature sintering to synthesize the desired compound (Figure 3a). This method is commonly used for large‐scale production and is practically applied in the industry because of its simplicity, affordability, and environmental friendliness. Nonetheless, the solid‐state method may cause poor sample consistency, which hinders its commercialization potential. The process typically involves grinding raw materials to increase their specific surface area for activation before sintering. The represented particles are generally agglomerated and heterogeneous (Figure 3b), adversely affecting the electrochemical performance of NFPP.

A straightforward two‐step solid‐state method was employed to synthesize NFPP [32]. Initially, Na_4_P_2_O_7_, Fe_2_C_2_O_4_·2H_2_O, and NH_4_H_2_PO_4_ were ball‐milled with acetone for 24 h, dried, and sintered at 300°C for 6 h. Subsequently, the calcined powders were regrounded, pelletized, and sintered at 500°C for 12 h to produce the final NFPP sample. Additionally, thermal stability analysis indicates that NFPP tends to decompose into two components (NaFePO_4_ and Na_2_FeP_2_O_7_) above 500°C. In another study, soot was added to raw materials to prepare NFPP [50]. The mechanical activation process was performed for 5 min using high‐energy planetary milling at 900 rpm, with a sintering process at 400–600°C for 4 h. This final product exhibited an initial discharge of 104 mAh g^−1^ at 0.2C and maintained 79% of its capacity after 50 cycles. These findings highlight the potential of NFPP and suggest additional work to enhance its performance further.

NFPP with porous structures can be fabricated through the solid‐state method, enhancing the electrochemical kinetics of electrons and ions. A porous structure NFPP‐P was synthesized using NH_4_H_2_PO_4_, FeC_2_O_4_·2H_2_O, NaHCO_3_, and lauric acid (C_12_H_24_O_2_) as raw material, in which lauric acid was used as carbon source and surfactant [51]. After mixing raw materials with absolute ethyl alcohol in ball milling at 600 rpm for 6 h, the precursor was dried and heated at 500°C for 12 h, resulting in the NFPP‐P. Notably, NFPP‐P exhibits an initial discharge capacity of 124.5 mAh g^−1^ at 0.1C with a capacity retention of 93.98% at 10C after 5000 cycles. In contrast, NFPP maintained only 92.1% at 10C after 1800 cycles, highlighting the significant impact of porous morphology. The porous NFPP‐P achieves high capacity and excellent cycle stability, proving the effectiveness of its structure.

The solid‐state method is cost‐effective and scalable, making it ideal for NFPP production. Despite sample consistency and particle uniformity issues, recent advancements in porous structure design have significantly enhanced electrochemical performance and capacity retention, suggesting a promising avenue for further refinement.

### 3.2 Spray‐Drying Method

The spray‐drying method involves atomizing a liquid feed into hot drying mediums to produce fine particles (Figure 3c). This technique can achieve high production capacity and excellent product quality, making it widely used in industrial production. Compared to the solid‐state method, spray drying can regulate the morphology and structure of NFPP by controlling process parameters, resulting in a final product with good consistency (Figure 3d). Despite these advantages, the spray‐drying method is more costly and energy‐intensive, which may limit its widespread use in certain cases.

A spray‐drying method was employed to synthesize NFPP@rGO using Fe(NO_3_)_3_·_9_H_2_O, NaH_2_PO_4_·2H_2_O, and C_6_H_8_O_7_·H_2_O as raw materials [48]. The NFPP@rGO exhibited a capacity of 128 mAh g^−1^ at 0.1C, a rate capability of 35 mAh g^−1^ at 200C, and maintained 62.3% of its capacity after 6000 cycles at 10C. Another study designed a green synthesis approach for Na_3_Fe_2_(PO_4_)(P_2_O_7_)@rGO, and all the atoms were utilized [52]. Sand milling and sonication ensure uniform mixing, while spray drying provides consistent particle size, enhancing conductivity and ion transport. Calcination at 500°C integrates rGO, boosting stability and conductivity. This results in a capacity of 105.5 mAh g^−1^ at 0.2C, 72.4% retention after 8000 cycles at 20C, and 42.4 mAh g^−1^ at 100C, demonstrating the method's efficiency and commercial potential. This environmentally friendly and low‐cost synthesis method is a reference for future commercialization.

Porous and hollow spherical morphology can be built through the spray‐drying method. By providing a shorter pathway for Na^+^ ion migration and enhancing electron transport, the porous and hollow spherical design effectively manages the volume expansion of NFPP. HS‐NFPP/C was obtained using rust as the iron resource [53]. Despite 10 000 cycles, the HS‐NFPP/C can still retain an impressive 87.5% capacity at 10C and its reversible capacities reached 86.5, 78.6, 68.9, and 57.1 mAh g^−1^ at 1, 10, 50, and 100C. Another study prepared hollow sphere structured NFPP powders using Fe(NO_3_)_3_·9H_2_O, CH_3_COONa, NH_4_H_2_PO_4_, and C_2_H_2_O_4_·2H_2_O, and glucose as raw material [54]. The solutions with raw material were stirred, spray‐dried at 260°C, and sintered at 550°C for 10 h. The optimal sample has a discharge capacity of 107.7 mAh g^−1^ at 0.2C and retains a capacity of 92% after 1500 cycles. These works underscore the significance of morphology, providing insights for further optimization.

The spray‐drying method can produce high‐quality, consistent materials and allows precise control over morphology, making it suitable for industrial use. Nonetheless, it is more expensive and energy‐demanding than the solid‐state method. Its ability to create advanced structures like porous and hollow forms enhances ion transport and stability, highlighting its commercial potential.

### 3.3 Sol–Gel Method

The sol–gel method (Figure 3e) facilitates homogeneous mixing of precursors at the molecular or nanoscale level, typically yielding products with consistent quality. The distinct porous structure of the final product provides a high specific surface area, enhancing the electrochemical performance of NFPP (Figure 3f). Nonetheless, achieving precise control over material morphology and microstructure remains challenging. Generally, the sol–gel process for NFPP employs phosphates that partially decompose into pyrophosphate [36, 37, 46, 55] [25, 30, 31, 56]. The presence of impurities and the complexity of the system can make the sol–gel method more sensitive to conditions, requiring more precise control compared to other methods.

In the synthesis of NFPP/C nanocomposites, the sol–gel method has been employed to optimize the material's performance [36]. Iron metal powder and citric acid were mixed and stirred in 30 mL deionized water at 80°C for 15 h. NaH_2_PO_4_·2H_2_O and ethylene glycol were then added to this solution and oil bath at 120°C for 1 h. After drying at 80°C in air and ground, the precursor was calcined for 10 h to obtain the final product. Although the crystal structure of NFPP can be damaged above 600°C, forming the unwanted maricite NaFePO_4_ and Na_2_FeP_2_O_7_, excessive phosphate can promote the complete formation of pyrophosphate. This prepared NFPP/C‐500 can deliver a discharge capacity of 99 mAh g^−1^ at 0.2C. Another study synthesized NFPP using FeC_2_O_4_·2H_2_O, Na_4_P_2_O_7_, NH_4_H_2_PO_4_, and sucrose, examining the unique pyrophosphate environment [57]. Their research found that Fe‐oxalate coordination dominates at low pH, while Fe(II)‐citric acid coordination occurs at neutral pH, indicating that PH adjustments influence reactant complexation and reaction pathways. The cathode material produced at pH = 7 exhibited superior electrical performance compared to that made at pH = 3 with Na_4_P_2_O_7_. These studies highlight the importance of synthesis conditions and pH control in optimizing NFPP/C performance.

3D interconnected porous and nanoplate‐like morphology can be created through the sol–gel method. Porous materials possess extensive surface areas and numerous active sites, enhancing their mass transfer capabilities. 3DIP‐NFPP/C was synthesized using a combined sol–gel method and solid‐state calcination, resulting in NFPP‐550 with a discharge capacity of 104.6 mAh g^−1^ at 0.1C [58]. In another study, nanoplate‐like NFPP with an average particle size of 150 nm was synthesized using CH_3_COONa, (NH_4_)_3_PO_4_, glucose, iron(II) acetate, ethylenediaminetetraacetic acid, cetyltrimethylammonium bromide, and stearic acid as raw materials [36]. This composite exhibited 108.3 mAh g^−1^ discharge capacity at 0.1C and maintained 80.3 mAh g^−1^ at 20C. Building on recent findings, the sol–gel method can enhance NFPP materials by creating 3D porous and nanoplate‐like structures, which significantly boost their discharge capacities.

While the sol–gel method excels in achieving uniform mixing and enhancing surface area for better electrochemical performance, it faces challenges in controlling morphology precisely and is sensitive to impurities. Nonetheless, its capacity to generate distinct structures makes it a key player in advanced material synthesis.

### 3.4 Electrospinning Method

The electrospinning method (Figure 3g) has become an important way to synthesize 1D nanomaterials (Figure 3h) with specific structures and morphologies due to its simplicity, versatility, and affordability. The final product is formed by activating the polymer solution in a high‐voltage electrostatic field, creating a jet stream that rapidly solidifies in the flight. While effective at the laboratory scale, scaling up the electrostatic spinning method for industrial production remains challenging in terms of quality and reliability.

Nanoribbons and nanofibers are constructed via the electrospinning method, enhancing the exceptional electrochemical performance of NFPP. The NFPP nanoparticles incorporated into carbon nanoribbons were prepared through polyvinylpyrrolidone‐assisted electrospinning [59]. This morphology facilitates rapid electron transfer along a 3D highway and prevents nanoparticle aggregation. This composite delivers a capacity of 128.6 mAh g^−1^ at 0.1C and maintains 72% of its capacity after 5000 cycles at 50C. In another study, using the electrospinning method followed by pyrolysis, hierarchically carbon‐decorated nanofibers were designed [45]. These nanofibers form a 3D network, enhancing electron conductivity and Na storage kinetics. The prepared material achieved a reversible capacity of 118 mAh g^−1^ at 0.2C and maintained 79.6% of its capacity after 10 000 cycles at 10C. Future research may consider optimizing these structures and exploring advanced modifications, further improving the performance of NFPP.

The electrostatic spinning method is simple and versatile, ideal for creating 1D nanomaterials with precise structures. It enhances electrochemical performance but faces challenges in scaling up to industrial production while maintaining quality.

### 3.5 Solution Combustion Method

The solution combustion method is favored due to its rapid reaction rate, simplicity, and high product purity. Completing a reaction within minutes is a significant advantage of this approach. Nonetheless, the high‐temperature reactions require stringent equipment and may generate harmful gases, posing potential risks to the environment and operator safety.

The solution combustion method, used for bulk material synthesis and thin film preparation, reveals the excellent performance of NFPP [39]. Initially, NaH_2_PO_4_, Fe(NO_3_)_3_·9H_2_O, and C_6_H_8_O_6_ were dissolved into 50 mL distilled water and stirred at 120°C for 2 h. The reactants were subsequently ignited on the hot plate at 300°C, and the final product was obtained by grinding and annealing at 600°C for 12 h. Thin films were then deposited onto stainless steel substrates using pulsed laser deposition (PLD). Notably, this material delivers a capacity of 125 mAh g^−1^ at a low current rate of 2 µA cm^−2^ and 110 mAh g^−1^ at a high current rate of 10 µA cm^−2^. After 500 cycles, no notable capacity fading was observed, with the Coulombic efficiency remaining at 98%. Interestingly, when used in potassium‐ion batteries, NFPP synthesized by solution combustion approach can achieve a discharge capacity of 120 mAh g^−1^ at a robust 3.0 V [55]. Future research may explore combining solution combustion with other modification strategies for simple preparation and further optimization of NFPP's performance.

The solution combustion method stands out for its rapid reaction and high purity, making it ideal for efficient synthesis. Nonetheless, the high temperatures required can pose environmental and safety challenges. Despite these concerns, its ability to produce high‐performance materials quickly and effectively makes it a valuable technique in material synthesis.

### 3.6 Template Method

The template method utilizes template materials to control the microstructure of the final product, offering significant potential for producing materials with specific sizes and shapes. This method enables precise regulation over the pore structure and morphology, significantly enhancing the electrochemical performance. Despite all of the advantages, this method requires special chemical processing steps to use and remove templating agents, which increases production costs and complicates the overall synthesis process.

Nanospheres NFPP/C with adjustable carbon coating thickness and particle size were prepared via a template method (Figure 3i,j) [46]. F127, NH_4_H_2_PO_4_, Na_4_P_2_O_7_, and Fe(NO_3_)_3_·9H_2_O were dissolved in deionized water, followed by the addition of HCl and a phenolic resol ethanol solution, which was stirred for 2 h. This emulsion was then dried for 24 h and subjected to heat treatment to obtain the final product. The nanosizing enhances electrochemical kinetics by shortening transport pathways for ions and electrons. These NFPP/C nanospheres exhibit a high discharge capacity of 128.5 mA g^−1^ at 0.2C, with 63.5% capacity retention at 10C after 4000 cycles and an excellent reversible capacity of 79 mAh g^−1^ at 100C. Future research may consider prioritizing enhancing specific capacity for NFPP/C nanospheres, followed by additional modifications to exceed theoretical capacity.

The template method precisely shapes cathode materials, improving their electrochemical performance. Although managing templates adds complexity and cost, its ability to create tailored structures makes it valuable for material design.

Different synthesis methods offer distinct advantages and uniquely impact the properties and performance of NFPP. Nonetheless, these methods also present numerous challenges that need careful consideration, especially regarding cost and impurity issues (Table 1).

**TABLE 1:** Comparison of NFPP materials synthesized via different methods.

Synthesis method	Particle size	Carbon content, wt.%	Initial Coulombic efficiency (ICE), %	Specific capacity, mash g^−1^	Rate performance	Capacity retention, %	Key features/remarks
Solid state	1–10 μm	0–5	80–90	90–110	Moderate (≤10C)	70–85 (100–500 cycles)	High crystallinity, low cost, but large particle size limits kinetics
Sol–gel	100–500 nm	5–15	85–95	100–120	Good (20–50C)	80–95 (200–1000 cycles)	Fine particles, uniform carbon coating, improved kinetics
Spray drying	1–5 μm (secondary), nanosized primary	5–10	85–92	95–115	Good (≤20C)	80–90 (200–500 cycles)	Spherical morphology, scalable process, improved packing density
Hydrothermal/Solvothermal	50–300 nm	5–12	85–95	100–120	Excellent (50–100C)	85–95 (500–2000 cycles)	Controlled morphology, high surface area, enhanced ion transport
Electrospinning	Nanofibers (diameter ~100–300 nm)	10–20	85–95	100–115	Excellent (≤50C)	85–95 (500–1000 cycles)	1D conductive network, continuous electron pathways
Spray pyrolysis	Submicron to micron	5–10	80–90	90–110	Moderate–good (≤20C)	75–90 (200–500 cycles)	One‐step synthesis, scalable, but less control over defects

From a critical perspective, the electrochemical performance of NFPP is strongly governed by several key factors associated with synthesis methods, including particle size, crystallinity, phase purity, and carbon distribution. Methods that enable uniform particle size and intimate carbon coating generally result in improved rate capability and cycling stability. In contrast, approaches with limited control over morphology or phase composition often led to inferior performance. Therefore, precise control of microstructure and impurity phases is more critical than the choice of synthesis method itself.

From a practical perspective, the scalability of synthesis methods is a critical factor for the commercialization of NFPP cathode materials. Among the reported approaches, the solid‐state method and spray‐drying method are considered the most promising for large‐scale production. The solid‐state method offers advantages in terms of simplicity, low cost, and compatibility with existing industrial infrastructure; however, it often suffers from poor particle uniformity and limited control over morphology. In contrast, spray drying enables better control over particle size, morphology, and compositional uniformity, producing spherical and homogeneous materials suitable for industrial electrode fabrication. Despite its higher energy consumption and equipment cost, spray drying is widely regarded as a scalable and industrially viable technique. Other methods, such as sol–gel and electrospinning, provide superior control over microstructure and performance but are generally limited by higher complexity and lower throughput, making them less suitable for mass production at the current stage.

In summary, different synthesis methods offer distinct advantages for NFPP materials. The solid‐state method is cost‐effective and suitable for large‐scale production but provides limited control over particle morphology. Wet‐chemical approaches, such as sol–gel, enable better compositional uniformity and microstructure control, while spray drying offers a balance between scalability and morphology regulation. Advanced techniques, including electrospinning and template‐assisted methods, can further optimize nanostructures but are less practical for industrial‐scale production. Therefore, selecting an appropriate synthesis route requires balancing performance optimization and scalability.

## 4 Strategies for the Modification of Na_4_Fe_3_(PO_4_)_2_(P_2_O_7_)

**FIGURE 4:**
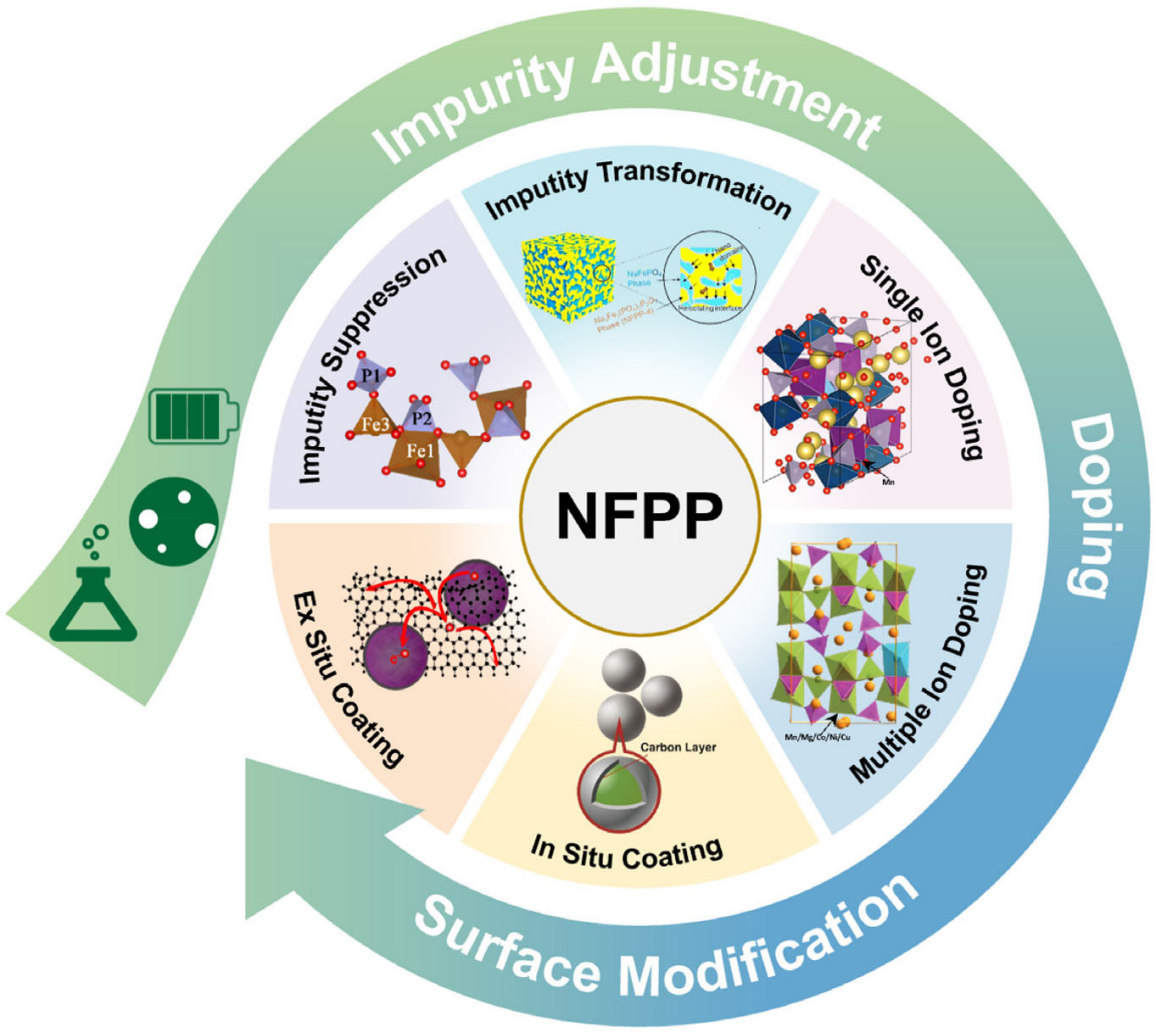
Strategies for the modification of Na_4_Fe_3_(PO_4_)_2_(P_2_O_7_).

The application of NFPP cathode materials is constrained by their large molecular mass and low electronic conductivity, which adversely affect their electrochemical performance, especially capacity and rate capability. Impurities are also a common issue during NFPP synthesis, negatively impacting the properties of NFPP. Therefore, handling these impurities and enhancing the electronic conductivity is a critical challenge for improving material performance. To date, researchers have explored various strategies to optimize the electrochemical performance of NFPPs, including doping, surface modification, and impurity modulation methods (Figure 4).

### 4.1 Impurity Adjustment

Impurities are a significant challenge in the NFPP system, hindering its overall electrochemical performance. Various modification strategies have been explored to address this issue, primarily focusing on two routes. One aims to suppress impurity generation by creating Fe defects or nonstoichiometric structures, while the other seeks to transform and utilize these impurities by constructing a heterostructure. Both strategies effectively address the impurity issue, offering novel solutions and providing innovative insights into NFPP research.

#### 4.1.1 Impurity Suppression Route

**FIGURE 5:**
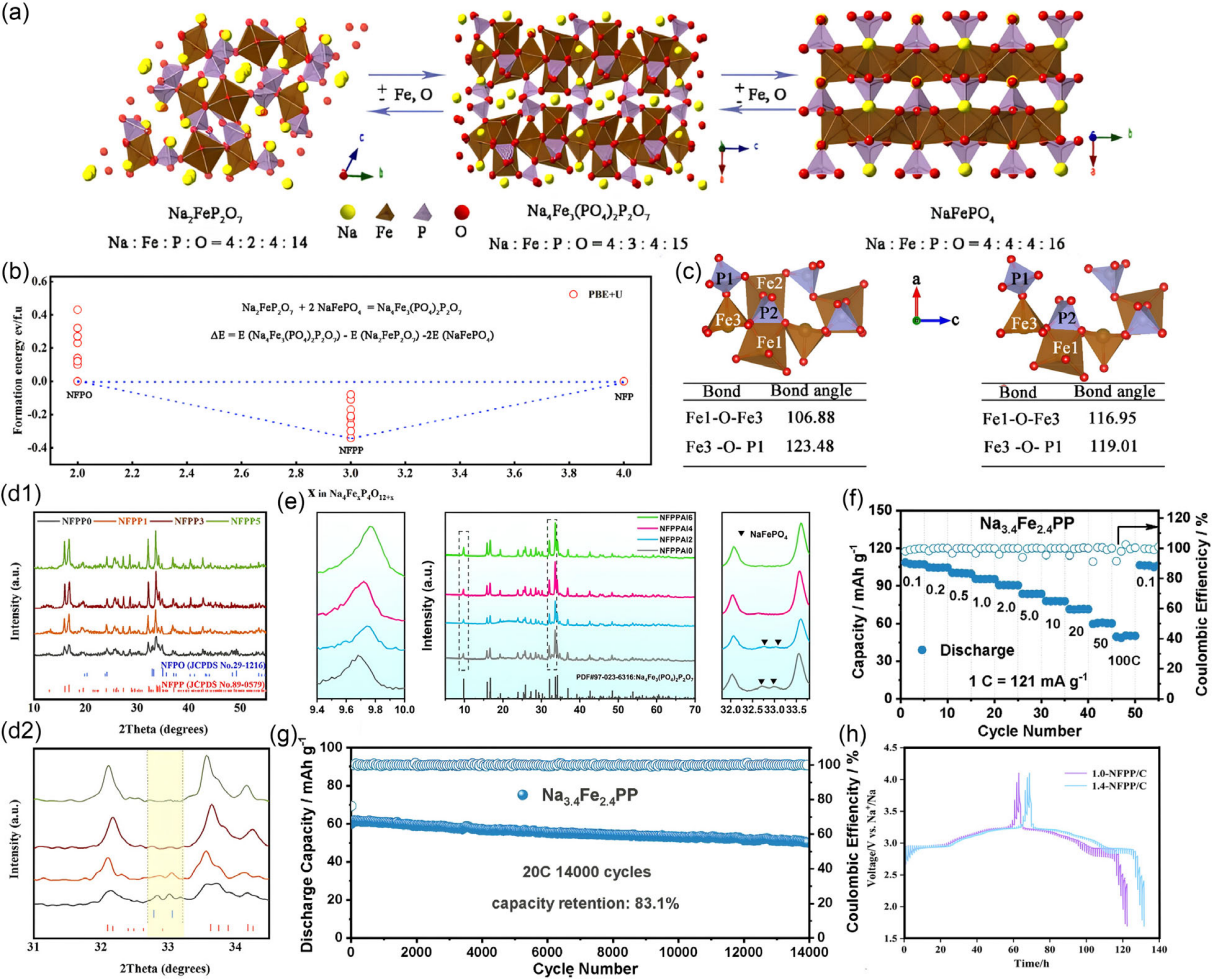
(a) Structural schematic and transition relation among NFPO, NFPP, and NFP [60]. Reproduced with permission [61]. Copyright 2022, American Chemical Society. (b) Formation energy of NFPP [60]. Reproduced with permission [61]. Copyright 2022, American Chemical Society. (c) Complete and Fe2 defective crystal structures of the NFPP phase [60]. Reproduced with permission [61]. Copyright 2022, American Chemical Society. (d) X‐ray diffraction patterns and enlarged patterns from 31° to 34.5° of NFPP [25]. Reproduced with permission [26]. Copyright 2022, Elsevier. (e) XRD patterns and local enlargements of NFPPAl0, NFPPAl2, NFPPAl4, and NFPPAl6 [62]. Reproduced with permission [64]. Copyright 2024, Elsevier. (f,g) Rate capability and long‐term stability of Na_3.4_Fe_2.4_(PO4)_1.4_P_2_O_7_ [61]. Reproduced with permission [63]. Copyright 2024, Wiley. (h) GITT curves of 1.0‐NFPP/C and 1.4‐NFPP/C [64]. Reproduced with permission [65]. Copyright 2024, American Chemical Society.

The primary impurities in NFPP products are maricite NaFePO_4_ (NFP) and Na_2_FeP_2_O_7_ (NFPO), which possess suboptimal electrochemical properties, hindering the performance of the integrated NFPP system. Figure 5a illustrates the transitions among NFPO, NFPP, and NFP systems. Most reported NFPP cathode materials are not pure phases but mixtures, complicating the system. The formation energy (Figure 5b) of NFPP was calculated (−0.38 ev/f.u.), demonstrating synthesis tends to result in NFPP formation [60]. Nonetheless, trace amounts of the maricite NFP impurities often persist in the final product [30]. NFPP remains stable below 600°C, but a complete transformation to maricite NFP occurs above 800°C due to the better thermodynamic stability of NFP [39]. Additionally, the rough selection of raw material can increase impurities due to the sensitivity of reactions.

Different proportions of components have been tried in the NFPP system to obtain the high pure phase and understand the mechanism of impurities. Studies have investigated the Na_*x* + 2_Fe_*x* + 1_(PO_4_)_*x*_P_2_O_7_ (*x* = 1,2,3,4) system by varying the molar ratio of (PO_4_)^3−^ to (P_2_O_7_)^4−^. It was discovered that a mixed phase appears when *x* is between 2 and 3, containing both NFPP and maricite NFP. Interestingly, some maricite NFP can be activated to become amorphous NFP, enhancing the capacity of mixed phases. To be more accurate, 17 Na_4_Fe_*x*_P_4_O_12+*x*_ samples with varied *x* were studied [60]. It was verified only three phases are present in the Na_4_Fe_*x*_P_4_O_12+*x*_ system and the possibility of producing a high‐purity Fe‐defective NFPP structure with *x* = 2.91 (Figure 5c). Although the theoretical capacity of NFPO electrodes can reach 98 mAh g^−1^, NFPO has been shown to hinder system integral electrochemical performance [63]. Specifically, increasing *x* values (2.00, 2.11, 2.25, 2.40, 2.55, 2.70, 2.86, and 2.9) in the Na_4_Fe_*x*_P_4_O_12+*x*_ system indicates a decrease in NFPO, resulting in an increase in discharge capacity from 85.7, 86.8, 88.4, 90.9, 93.2, 98.1, 105.0, to 110 mAh g^−1^. Another study also explored NFPP‐x (*x* = 3.00, 2.90, 2.85, 2.70) and examined the relationship between Fe concentration and the content of NFPP, NFP, and NFPO. Among these, NFPP‐2.85 demonstrated the best performance, achieving 111.8 mAh g^−1^ at 0.1C [65]. Future research may continue optimizing component ratios to achieve high purity and performance.

The optimal Fe content for achieving a high‐purity phase can be attained by incorporating a few Fe defects into the NFPP lattice [25]. Interestingly, X‐ray diffraction patterns in Figure 5d reveal that the NaFePO_4_ impurity decreases as Fe defects increase, with 3% Fe defect indicating the high‐purity phase Na_4_Fe_2.91_(PO_4_)_2_(P_2_O_7_). These defects in NFPP lower the bandgap and migration energy barriers, enhancing Na^+^ ion and electron conductivity. This pure‐phase Na_4_Fe_2.91_(PO_4_)_2_(P_2_O_7_) material exhibits outstanding long‐cycle stability over 10 000 cycles, a high discharge capacity of 110.9 mAh g^−1^ at 0.2C, and a rate performance (52 mAh g^−1^ at 100C). Additionally, appropriate doping can suppress impurity production and enhance electrochemical performance [62, 66, 67]. For instance, the characteristic peak of impurity NaFePO_4_ (Figure 5e) gradually decreased with the increased Al doping in NAFPP as reduced Fe content inhibits NaFePO_4_ generation. Likewise, pure NFPP was observed with 6% nickel substitution in another study [62]. The cooperative interaction of doping and Fe content adjustment may offer valuable insight into NFPP's modification. The possible charge compensation mechanisms in nonstoichiometric NFPP include Fe valence adjustment (Fe^2+^/Fe^3+^ redox), sodium vacancy formation, and local structural rearrangements.

Nonstoichiometric NFPP material can achieve high‐purity phases and exhibit remarkable electrochemical performance. Stoichiometric polyanions generally introduce impurities, restricting their commercial viability. A high‐purity Na_3.4_Fe_2.4_(PO_4_)_1.4_P_2_O_7_ cathode material was prepared through precise molecular structure design [61]. This nonstoichiometric material demonstrates excellent electrochemical performance, with a capacity of 50.5 mAh g^−1^ at 100C and a cycle life of over 14 000 cycles at 20C (Figure 5f,g). In another study, a pure‐phase Na_3.4_Fe_2.4_(PO_4_)_1.4_P_2_O_7_/C(1.4‐NFPP/C) material was developed using a spray‐drying method [64]. As shown in the galvanostatic intermittent titration testing (GITT) curves in Figure 5h, 1.4‐NFPP/C displayed a lower overpotential than 1.0‐NFPP/C during discharge, leading to faster redox kinetics. The 1.4‐NFPP/C exhibits a discharge capacity of 112.2 mAh g^−1^ at 0.1C, a rate performance reaching 70 mAh g^−1^ at 30C, and outstanding long‐term cycle performance, remaining 99.7% after 1000 cycles at 10C. It should be noted that beyond the stoichiometric system, there remains a significant research gap in nonstoichiometric modulation, which could address existing challenges and unlock new opportunities.

While the introduction of Fe defects and nonstoichiometry can effectively suppress impurity phase formation and, in some cases, enhance electrochemical activity, excessive defect concentrations may adversely affect structural stability. From a crystallographic perspective, a high density of defects (e.g., Fe vacancies, Na deficiencies, or antisite disorder) can induce significant lattice distortion and local strain accumulation, which may weaken the robustness of the polyanion framework. During repeated Na^+^ insertion/extraction, such structurally perturbed regions are more susceptible to irreversible rearrangement, potentially triggering phase transitions or partial amorphization. In addition, excessive defects may disrupt long‐range Na^+^ diffusion pathways, leading to increased polarization and deteriorated rate capability over extended cycling. There is therefore a critical balance between beneficial defect engineering and structural integrity: moderate defect concentrations can facilitate charge compensation and suppress parasitic phase formation, whereas overaccumulation may accelerate capacity fading and compromise long‐term cyclability. Accordingly, careful control of defect chemistry is essential to optimize both electrochemical performance and structural durability in NFPP systems.

Other emerging approaches are also effective for suppressing the impurities. Small amounts of LaBr_3_ were added as an additive to the synthesis of Na_4−2*x*_Fe_3–1.5*x*_La_*y*_(PO_4−*x*_Br_*x*_)_2_P_2_O_7_ with dual‐site defects. In this composite, the introduction of Fe/Na dual‐site defects narrows the bandgap and creates efficient pathways for Na^+^ ions, enhancing the rate capability and stability of NFPP. The cathode achieves impressive results, delivering 55.2 mAh g^−1^ at 50C and retaining 93% of its capacity after 2000 cycles at 10C [68]. Another study developed Na_4−*x*_Fe_3_(PO_4_)_2_P_2_O_7_ to achieve a pure‐phase NFPP. This approach created Na ion vacancies, which were filled by iron ions, resulting in an optimal Na_4−*x*_Fe_3_(PO_4_)_2_P_2_O_7_ with a specific capacity of 127.2 mAh g^−1^ [69]. Adding LaBr_3_ and engineering Na ion vacancies are promising methods to improve NFPP purity and performance, leading to better electrochemical applications.

To sum up, these innovative strategies for impurity suppression and phase optimization in NFPP materials not only enhance electrochemical performance but also lay the groundwork for future breakthroughs. By refining these methods, we can unlock new potentials in energy storage technologies, driving advancements in battery efficiency and sustainability.

#### 4.1.2 Impurity Transformation Route

**FIGURE 6:**
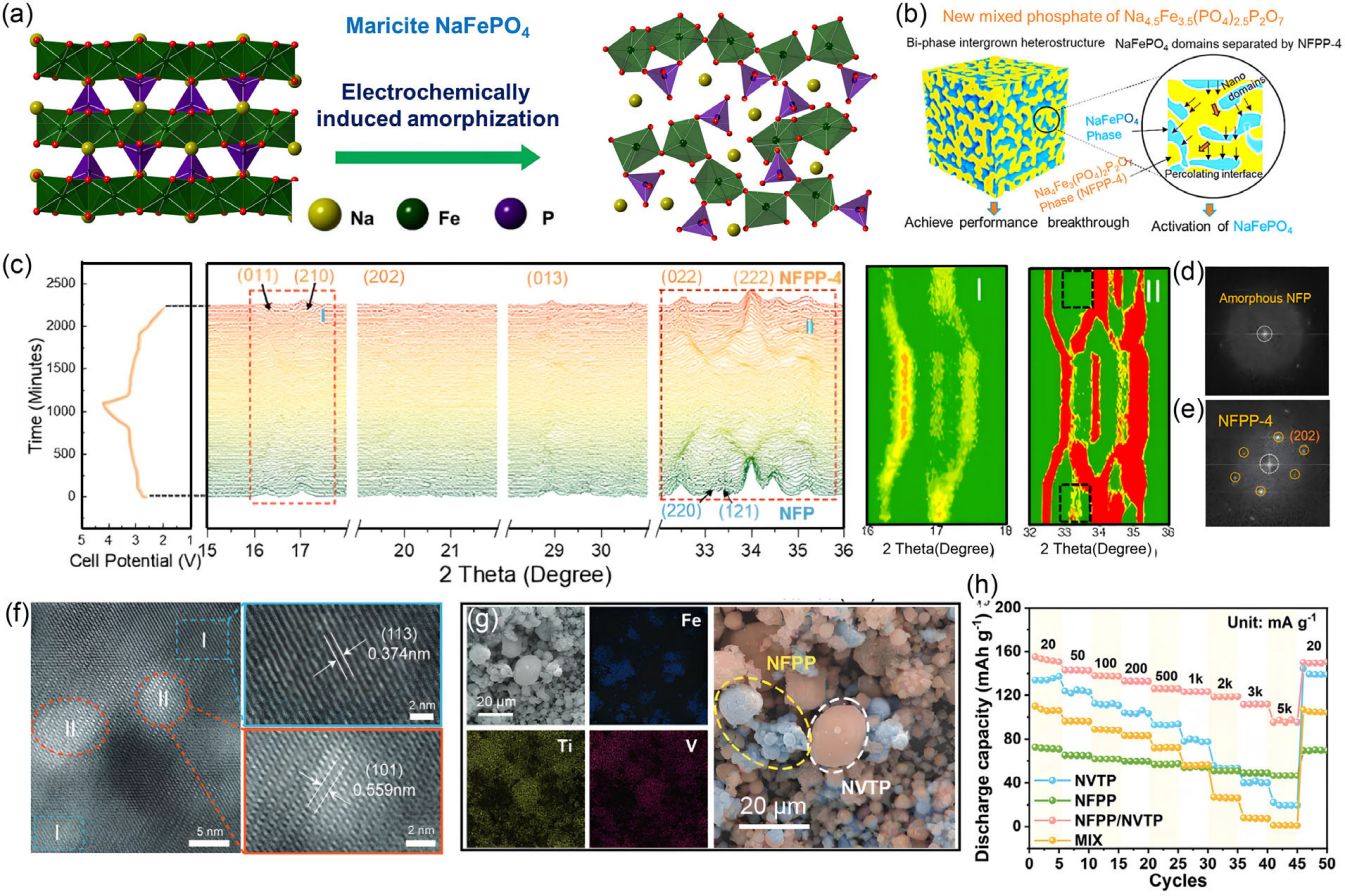
(a) Schematic diagram illustrating the structural evolution of maricite‐type NaFePO_4_ during electrochemically induced amorphization [72]. (b) Schematic illustration of the NFPP‐4.5 cathode with a two‐phase intergrown heterostructure of NFP and NFPP‐4 [49]. Reproduced with permission [51]. Copyright 2024, American Chemical Society. (c) Operando XRD patterns of NFPP‐4.5 electrode, featuring 2D contour plots within a selected 2θ range and the corresponding charge/discharge curves during the first cycle [49]. Reproduced with permission [51]. Copyright 2024, American Chemical Society. (d,e) Corresponding FFT patterns in crystalline and amorphous regions in NFPP‐4.5 [49]. Reproduced with permission [51]. Copyright 2024, American Chemical Society. (f) HR‐TEM illustrations of Na_3_V_2_ (PO_4_)_3_‐Na_3_Fe_2_(PO_4_)P_2_O_7_ [73]. Reproduced with permission [74]. Copyright 2022, Wiley. (g) EDS mapping and SEM image. (h) Rate performance of Na_4_Fe_3_(PO_4_)_2_(P_2_O_7_)/Na_2_VTi(PO_4_)_3_ [75]. Reproduced with permission [76]. Copyright 2024, Wiley.

The crystalline structure of NaFePO_4_ includes a maricite phase and an olivine phase [70]. As an independent electrode, the olivine NFP cathode can achieve a large theoretical capacity of 154 mAh g^−1^ and an operational potential of 2.9 V [8]. Nonetheless, maricite NaFePO_4_ is the thermodynamically stable phase [71]. Olivine NaFePO_4_ can only be synthesized through specialized methods, initially involving the electrochemical delithiation of LiFePO_4_ and electrochemical sodiation of FePO_4_ to form NaFePO_4_. While maricite NaFePO_4_ is generally an electrochemically inactive electrode for SIBs, nanosized maricite NaFePO_4_ can demonstrate remarkable electrochemical activity because of structure transformation into the amorphous phase (Figure 6a). The potential to activate maricite NaFePO_4_ into amorphous NaFePO_4_ reveals a potential route for impurity transformation.

Nanosized maricite NaFePO_4_ can be stably contained as the shell of Na_4_Fe_3_(PO_4_)_2_(P_2_O_7_), promoting its electrochemical activation. A core‐double shell Na_4_Fe_3_(PO_4_)_2_(P_2_O_7_)@NaFePO_4_@C was prepared using a versatile sol–gel fabrication method followed by heat treatment [74]. Notably, the maricite NaFePO_4_ shell maintains a thickness of 15 nm, enabling its electrochemical activation. The joint effort of the activated NaFePO_4_ and the rapid 3D carbon shell allows Na_4_Fe_3_(PO_4_)_2_(P_2_O_7_)@NaFePO_4_@C to deliver an exceptional discharge capacity of 136 mAh g^−1^ at 0.1C, beyond the theoretical capacity of NFPP. Additionally, this material demonstrates outstanding cycling stability with no degradation for over 3000 cycles at 10C and an excellent rate capacity of 68 mAh g^−1^ at 100C. This innovative strategy highlights the potential to utilize NaFePO_4_, indicating the possibility of using impurities.

While nanosized NaFePO_4_ can be activated as a shell of NFPP, maricite‐type NaFePO_4_ can also intergrow with Na_4_Fe_3_(PO_4_)_2_(P_2_O_7_) phase to form a heterostructure. Na_4.5_Fe_3.5_(PO_4_)_2.5_P_2_O_7_ composites with a heterostructure were prepared using sol–gel method (Figure 6b) [49]. As shown in the operando XRD patterns in Figure 6c, the typical shoulder peaks of maricite NFP's (220) and (121) faces disappear in the fully discharged state at 2.0 V, indicating a spontaneous amorphization transition of the NFP phase. The FFT electron diffraction patterns in Figure 6d,e further confirm the high‐crystallinity NFPP‐4 phase and the amorphous NFP structure. Despite 11 000 cycles at 20C, the capacity of Na_4.5_Fe_3.5_(PO_4_)_2_P_2_O_7_ remains at 88%. Notably, this composite exhibits a reversible capacity of over 130 mAh g^−1^ and an energy density of 400 W h kg^−1^, the highest among iron‐based polyanionic cathodes for SIBs.

Besides heterogeneous structures with maricite NaFePO_4_, other heterogeneous structures can also affect impurity production while enhancing the performance of NFPP. Notably, the intergrowth structure helps improve the purity and crystallization of NFPP. A heterogeneous Na_3_V_2_(PO_4_)_3_‐Na_3_Fe_2_(PO_4_)P_2_O_7_ composite with a 1:1 molar ratio was prepared using a site preparation strategy [73]. In Figure 6f, areas with different lattice spacings of 0.374 and 0.559 nm, correspond to the (113) plane of the Na_3_V_2_(PO_4_)_3_ phase and (101) plane of the Na_3_Fe_2_(PO_4_)(P_2_O_7_) phase, indicating successful preparation of the heterogeneous products. This composite delivers a reversible capacity of 113.1 mAh g^−1^ at 0.1C and an energy density of 357 W h kg^−1^. The preparation of heterogeneous composites effectively enhances NFPP performance, and future research can focus on exploring more effective cathode material combinations.

Another heterogeneous material, Na_4_Fe_3_(PO_4_)_2_(P_2_O_7_)/Na_2_VTi(PO_4_)_3_ (NFPP/NVTP), was prepared through the spray‐drying method [75]. Two distinct crystal orientations with a clear region interface and tight contact were discovered. Additionally, Figure 6g provides direct evidence of the cross‐distribution of NFPP and NVTP in the heterostructure, as the abundance of Fe coincides with the scarcity of V and Ti. It should be mentioned that the NFPP/NVTP cathode delivers a high capacity of 155.3 mAh g^−1^ at 20 mA g^−1^ (Figure 6h) and an outstanding energy density of up to 417 W h kg^−1^. This material demonstrates a capacity retention of 82.9% after 2500 cycles at 1 A g^−1^. While these heterogeneous structures effectively improve the energy density of NFPP, a significant knowledge gap remains to be explored.

The impurity issue is a major distinction between Na_4_Fe_3_(PO_4_)_2_(P_2_O_7_) and other polyanionic materials, introducing new variables to the synthesis process and increasing the complexity of the NFPP system. Both impurity suppression and transformation strategies can enhance the electrochemical performance of NFPP. Nonetheless, impurity control can be integrated with other modification strategies to enhance the electrochemical performance of NFPP while controlling the impurity content.

### 4.2 Doping

**FIGURE 7:**
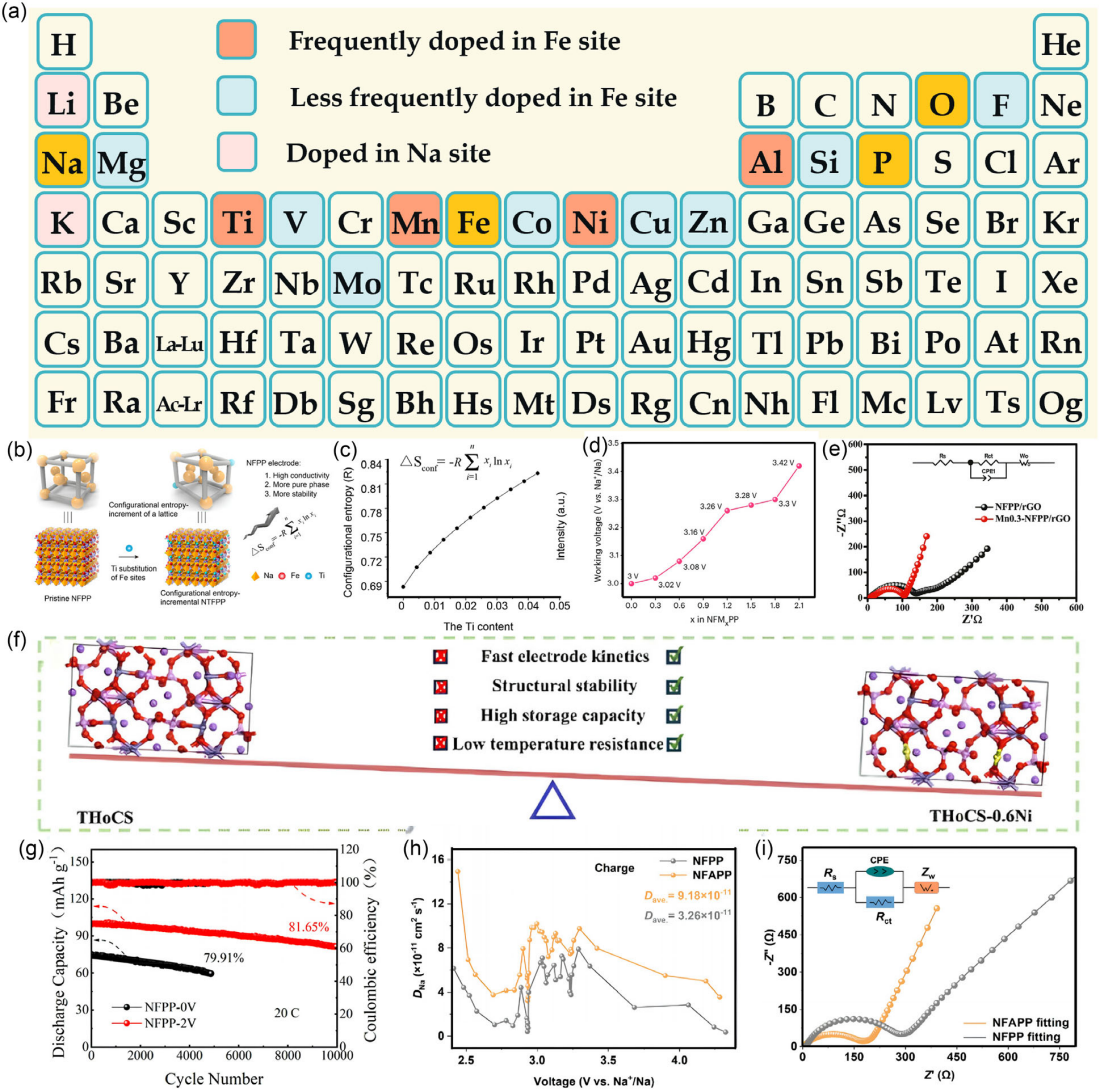
(a) Statistical diagram of doped elements. (b) Design concept illustrating entropy increase with Ti doping in NFPP [76]. Reproduced with permission [77]. Copyright 2024, American Chemical Society. (c) Calculation of configurational entropy upon Ti substitution at Fe sites in the NFPP lattice [76]. Reproduced with permission [77]. Copyright 2024, American Chemical Society. (d) Operating voltages of NFPP and NFM_*x*_PP at 0.1C in Na half‐cells [78]. Reproduced with permission [79]. Copyright 2024, Wiley. (e) Nyquist plots from EIS and fitting results for NFPP/rGO and Mn0.3‐NFPP/rGO [77]. Reproduced with permission [80]. Copyright 2022, Elsevier. (f) Comparison between THoCS and THoCS‐0.6Ni samples [79]. Reproduced with permission [81]. Copyright 2024, Wiley. (g) Cycle performance of Na_3.94_Fe_2.94_V_0.06_(PO_4_)_2_(P_2_O_7_) material at 20C over 10 000 cycles [66]. Reproduced with permission [68]. Copyright 2024, American Chemical Society. (h) EIS spectra for NFPP and NFAPP cells [80]. Reproduced with permission [82]. Copyright 2024, Elsevier. (i) Na^+^ ion diffusion dynamics of NFPP and NFAPP at various voltages based on GITT measurement [80]. Reproduced with permission [82]. Copyright 2024, Elsevier.

As an effective modification strategy, doping can help enhance the electrochemical performance of NFPP materials by adjusting the crystal structure. To date, researchers have investigated a wide range of elements as dopants including titanium (Ti), manganese (Mn), nickel (Ni), aluminum (Al), magnesium (Mg), cobalt (Co), molybdenum (Mo), copper (Cu), zinc (Zn), fluorine (F), vanadium (V), silicon (Si), and so on (Figure 7a).

#### 4.2.1 Single‐Ion Doping

Single‐cation doping, due to its simplicity and ease of control, has emerged as a crucial strategy for enhancing the electrochemical performance of NFPP materials. This approach can significantly optimize the electrochemical properties of NFPP cathode materials in SIBs, including stabilizing the operating voltage plateau, boosting the reversible capacity, and improving cycling stability.

The nontoxic and safe properties of titanium (Ti) have made it one of the most popular active elements. Titanium doping enhances the electrochemical activity of NFPPs by improving their electronic conductivity. The synthesized Na_4_Fe_2.98_Ti_0.01_(PO_4_)_2_(P_2_O_7_) material exhibits a discharge capacity of 112.5 mAh g^−1^ at 0.1C and a capacity of 97.2 mAh g^−1^ at 10C over 5000 cycles [81]. During the de/sodiation process, a volumetric change of merely 2.98% was recorded, signifying an improved reversibility of the crystal lattice. As shown in Figure 7b,c, the 5% Ti‐substituted lattice (NTFPP‐0.05) causes entropic augmentation, enhancing electronic conductivity and inhibiting the impure phases of NaFeP_2_O_7_ and NaFePO_4_. The optimal NTFPP‐0.05 composite demonstrates a high initial discharge capacity (118.5 mAh g^−1^ at 0.1C) [76]. Another study indicates that the synthesized Ti‐NFPP/C cathode demonstrates outstanding rate performance, achieving 66.7 mAh g^−1^ at 30C, and exhibits remarkable cycling stability with a capacity retention of 93.8% after 2000 cycles at 10C [82]. These findings highlight the potential of Ti doping to enhance NFPP performance.

Manganese (Mn), with the features of being environmentally friendly and cost‐effective, is widely used in doping strategies. The operating voltage of NFM_*x*_PP was enhanced by increasing the Mn content (Figure 7d). Introducing Mn (0.6 ≤ *x*  ≤ 1.2) into NFM_*x*_PP can also weaken the Fe–O bonding interaction, allowing full utilization of the Mn^3+^/Mn^2+^ redox couple. A series of Na_4_Fe_3−*x*_Mn_*x*_(PO_4_)_2_(P_2_O_7_) cathode have been studied revealing that the NFM_1.2_PP cathode exhibited a high operating voltage of approximately 3.3 V with a reversible capacity of 109.2 mAh g^−1^ [78]. Mn doping at the Fe site decreases Na^+^ ion diffusion energy barrier and the electronic bandgap, thereby synchronizing rapid electron and Na^+^ ion mobility. In another investigation, a 5% Mn‐doped Na_4_Fe_2.85_Mn_0.15_(PO_4_)_2_(P_2_O_7_) composite was prepared, exhibiting ultralong‐lasting cyclability with 88.1% capacity retention over 10 000 cycles at 50C and an impressive rate capability of 42.7 mAh g^−1^ at 200C [47]. The optimal Na_4_Fe_2.7_Mn_0.3_(PO_4_)_2_(P_2_O_7_)/rGO provides an initial discharge capacity of 131.5 mAh g^−1^ at 0.1C and excellent rate performance of 70.2 mAh g^−1^ at 50C [77]. The Mn0.3‐NFPP/rGO demonstrates superior ion conductivity, which is advantageous for rapid ion diffusion. It maintains a discharge capacity of 85.3 mAh g^−1^ at 0.2C even at −20°C, along with good rate performance (Figure 7e). Additionally, Na_4_Fe_2.9_Mn_0.1_(PO_4_)_2_(P_2_O_7_)@C composites was prepared by mechanochemically assisted approach, releasing 119.6 mAh g^−1^ at 0.1C and 84.8% after 3000 cycles at 10C [43]. Future research on Mn doping can explore new composites and optimize doping levels for better performance.

Doping with nickel (Ni) heteroatoms can enhance intrinsic electron conductivity and refine the Na^+^ ion diffusion pathway and energy barrier, facilitating rapid charging and Na storage at low temperatures (Figure 7f). A hollow core‐shelled Na_4_Fe_2.4_Ni_0.6_(PO_4_)_2_(P_2_O_7_) with minimal void space has been developed, exhibiting a superior rate capability of 86.4 mAh g^−1^ at 25C and exceptional cycling stability at −20°C, maintaining a capacity of 43.6 mAh g^−1^ after 2500 cycles at 5C [79]. Additionally, Ni can stabilize the crystal structure and expand the Na^+^ ion migration. The Na_3_Fe_1.9_Ni_0.1_(PO_4_)(P_2_O_7_) was synthesized through the sol–gel method delivering a discharge capacity of 100.7 mAh g^−1^ at 0.1C and excellent cycling stability throughout 5000 cycles at 10C [83]. Interestingly, Ni substitution can also inhibit electrochemical inert maricite‐NaFePO_4_ impurity generation [67]. The optimal Na_4_Fe_2.82_Ni_0.18_(PO_4_)_2_(P_2_O_7_)@C‐N cathode delivers an exceptionally high discharge capacity of 128.4 mAh g^−1^ at 0.2C, outstanding rate performance of 62.5 mAh g^−1^ at 50C, and impressive long‐term cycling stability, maintaining approximately 83% capacity retention after 3000 cycles at 10C. These advancements underscore the efficacy of Ni doping in optimizing the performance of NFPP.

Tiny amounts of vanadium (V) doping can also significantly improve the electrochemical performance of NFPP without excessive costs. NFPP doped with vanadium demonstrates lower barriers for sodium‐ion mobility and increased conductivity, resulting in enhanced electrochemical performance. The Na_3.94_Fe_2.94_V_0.06_(PO_4_)_2_(P_2_O_7_) exhibits a reversible capacity of 123.4 mAh g^−1^ at 0.1C [66]. Even at 20C, this composite maintains a reversible capacity of 99.6 mAh g^−1^ and maintains 81.65% capacity after 10 000 cycles (Figure 7g). Another study on vanadium doping confirmed that the appropriate introduction of V^3+^ can suppress the formation of NFP impurities, resulting in Na_3.95_Fe_2.95_V_0.05_(PO_4_)_2_P_2_O_7_ composite with a capacity of 114 mAh g^−1^ at 0.1C [84]. While cost reduction is a primary driver for the commercialization of NFPP, incorporating a small amount of V proves feasible if it substantially enhances electrochemical performance.

Aluminum (Al) doping has garnered extensive attention as a dopant for NFPP. Doping Al elements can effectively enhance NFPP's electronic conductivity, as illustrated in Figure 7h. NFAPP exhibits higher Na^+^ ion diffusion coefficients (*D*_Na_) compared to NFPP at the same voltage, indicating that Al substitution promotes Na^+^ ion dynamics (Figure 7i). A microporous Na_3.9_Fe_2.9_Al_0.1_(PO_4_)_2_(P_2_O_7_) (NFAPP) has been developed, demonstrating an overall volume change of around 3.5% and maintaining 85.1% capacity over 10 000 cycles at 50C [80]. Na_4_Fe_2.94_Al_0.04_(PO_4_)_2_(P_2_O_7_)/C was synthesized via a straightforward ball‐milling technique [62]. The average length of the Al–O bond is a little shorter than that of the Fe–O bond, which contributes to structure stability. By minimally substituting Al^3+^ for Fe^2+^ in the NFPP structure, Fe defects are introduced, which impede the NaFePO_4_ formation and improve Na^+^ ion diffusion kinetics and conductivity, resulting in Na_4_Fe_2.94_Al_0.04_(PO_4_)_2_P_2_O_7_/C cathode a discharge capacity of 128.1 mAh g^−1^ at 0.2C. Another Al doping study emphasizes the impact of low‐energy 3p orbitals and transition metal vacancies. These low‐energy p‐orbitals can lower the conduction band and enhance metal–oxygen covalent bonds, resulting in a reduced bandgap and a reinforced crystal structure [85]. This leads to Na_4_Fe_2.85_Al_0.1_(PO_4_)_2_(P_2_O_7_) achieving exceptional cycling stability, enduring over 10 000 cycles without significant degradation.

Magnesium (Mg), copper (Cu), and molybdenum (Mo) have been used in NFPP doping due to their distinct roles [86, 87]. The incorporation of Cu into NFPP enhances the local electron density and reduces the bandgap. Specifically, the synthesized Na_4_Fe_2.7_Cu_0.3_(PO_4_)_2_P_2_O_7_ achieves a discharge capacity of 119.01 mAh g^−1^ at 1C and retains 82.76% of its capacity after 3000 cycles at 20C [88]. Additionally, pure‐phase Mo^6+^‐doped Na_4_Fe_3*−x*_Mo_*x*_(PO_4_)_2_(P_2_O_7_)/C was synthesized via spray drying and annealing. DFT calculations demonstrate that Mo^6+^ doping enhances the intrinsic electron conductivity of Mox‐NFPP by facilitating the transition of electrons from the valence band to the conduction band. At 0.1C, the Mo0.10‐NFPP cathode delivers an initial discharge capacity of up to 123.9 mAh g^−1^, nearly its theoretical capacity [67]. Even at a high rate of 10C, it achieves a substantial discharge capacity of 86.09 mAh g^−1^ and retains 96.18% of its capacity after 500 cycles. The successful application of Mg, Cu, and Mo doping in NFPP highlights a clear path forward for optimizing its performance in advanced energy storage devices.

**FIGURE 8:**
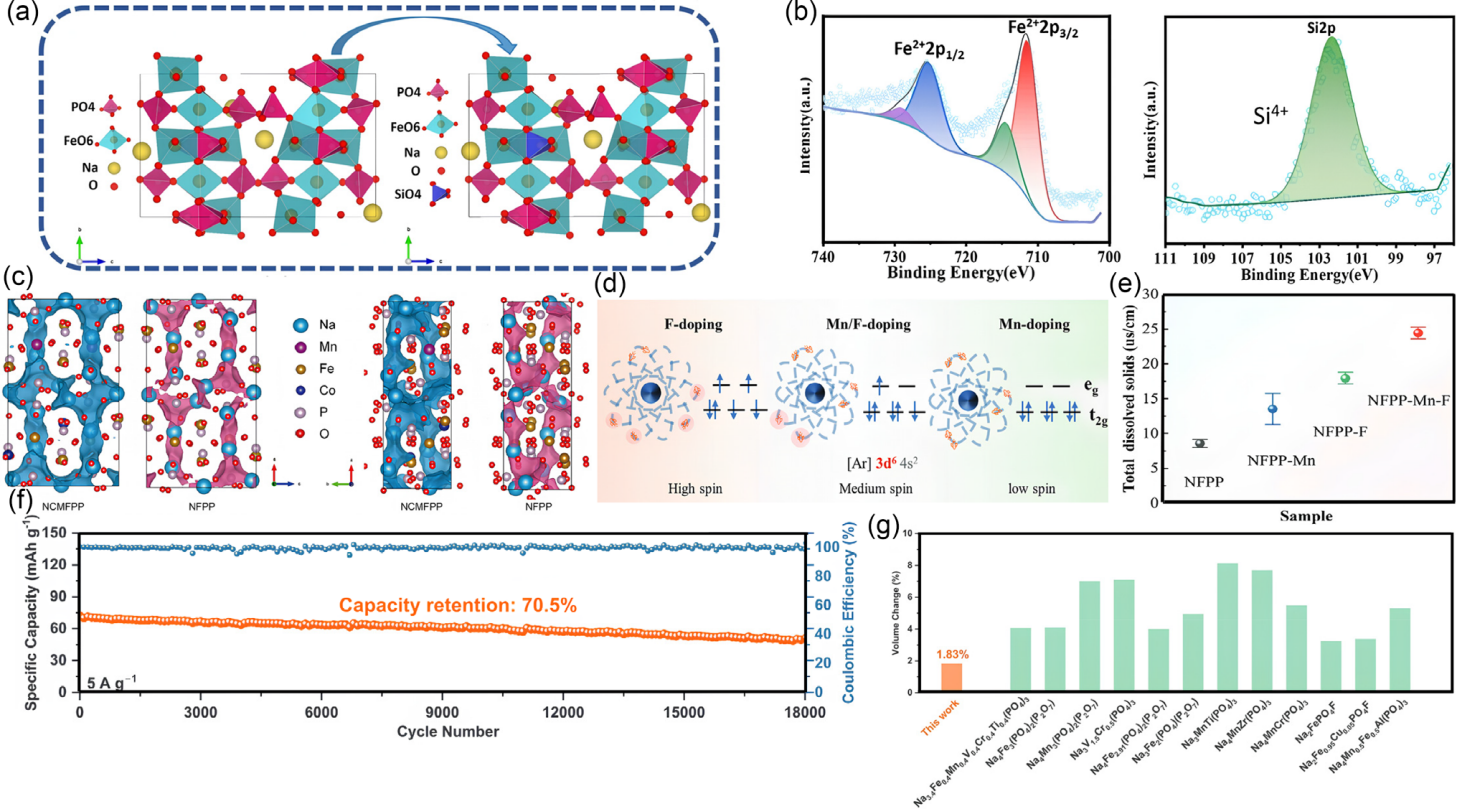
(a) Mechanism of (SiO_4_)^4−^ doping into NFPP [89]. Reproduced with permission [92]. Copyright 2023, American Chemical Society. (b) XPS spectra of NFPP‐Si0.05 sample showing Fe2p and Si2p peaks [89]. (c) Na^+^ ion diffusion pathways in NCMFPP and NFPP analyzed using the BVEL method [91]. Reproduced with permission [92]. Copyright 2024, Elsevier. (d) Schematic illustration of Fe spin transition. (e) Four‐point probe conductivity measurements for NFPP, NFPP‐Mn, NFPP‐F, and NFPP‐Mn‐F [92]. Reproduced with permission [93]. Copyright 2024, Wiley. (f) Long‐term cycling stability of HE‐NFPP at 5 A g^−1^ [90]. Reproduced with permission [94]. Copyright 2024, Wiley. (g) Volumetric variation comparison between HE‐NFPP and other sodium‐ion cathode materials [90]. Reproduced with permission [92]. Copyright 2024, Wiley.

Anionic substitution is an effective strategy to expand the modification strategies for NFPP materials. The electrochemical properties can be fine‐tuned by replacing anions in the NFPP structure, providing a more favorable migration path for Na^+^ ions. As illustrated in Figure 8a, the substitution of the (PO_4_)^3−^ group with the (SiO_4_)^4−^ group enhances diffusion kinetics and electronic conductivity, while maintaining structural integrity [89]. The incorporation of (SiO_4_)^4−^ into the NFPP matrix has been confirmed by XPS analysis as shown in Figure 8b. By substituting at a nonactive site, the electrochemical performance is improved without affecting the theoretical capacity. Consequently, this (SiO_4_)^4−^‐doped Na_4_Fe_3_(PO_4_)_2_(P_2_O_7_) exhibits a capacity of 119.4 mAh g^−1^ at 0.1C. Another study prepared the NASICON‐Na_4_Fe_3_(PO_4_)_1.9_(SiO_4_)_0.1_P_2_O_7_ was prepared. The substitution of SiO_4_^4−^ slightly enlarges the crystal lattice, thereby widening the Na^+^ diffusion channel, resulting in NFPP‐Si0.1 exhibiting a high rate capability of 77.6 mAh g^−1^ at 50C with a retention rate of 79.4% after 7000 cycles at 10C. These findings underscore the substantial potential of anionic substitution and offer valuable insights into the modification of NFPP materials.

#### 4.2.2 Multiple‐Ion Doping

Innovative doping techniques, including dual and high‐entropy strategies, are at the forefront of enhancing the electrochemical performance of NFPP materials. These approaches improve capacity and stability and open new avenues for developing advanced energy storage systems.

Dual doping is a strategy to optimize the electrochemical properties of NFPP by simultaneously introducing two doping elements into their structures. This approach can induce a synergistic effect, significantly enhancing the overall performance of the material through interactions between different doping elements [93]. Dual‐cation doping of cobalt (Co) and manganese (Mn) effectively increases the voltage and electrochemical capacity of NFPP. Na_4_Co_0.5_Mn_0.5_Fe_2_(PO_4_)_2_(P_2_O_7_) is prepared to facilitate a redox reaction involving 3.5 electrons during the charge/discharge process [91]. NCMFPP exhibits a more consistent Na^+^ ion diffusion path than NFPP and a broader Na^+^ ion diffusion channel along the *c*‐axis direction, as shown by the bond‐valence energy landscape (BVEL) analysis in Figure 8c. It was found that the NCMFPP provides a high initial discharge capacity of 139 mA g^−1^ at 0.1C with rate capacity of 75 mA g^−1^ at 10C.

In another study, a novel Na_4_Fe_2.96_Mg_0.02_Cu_0.02_(PO_4_)_2_P_2_O_7_ cathode material was synthesized. The dual doping of Mg and Cu significantly enhanced the electronic conductivity by narrowing the bandgap, achieving a discharge capacity of 112.3 mAh g^−1^ [92]. As shown in Figure 8d, dual doping with Mn and fluorine (F) can modulate the electronic structure, thus enhancing electronic conductivity. The material's bandgap was reduced from 1.01 to 0.80 eV, while its electronic conductivity increased from 8.5 to 24.4 µS cm^−1^, resulting in competitive electrochemical performance (Figure 8e) [92]. This composite demonstrates excellent rate performance, achieving 121.0 mAh g^−1^ at 0.1C and 104.9 mAh g^−1^ at 5C, and retaining 88.5% of its capacity after 1000 cycles at 1C. Future research can explore more dual‐doping combinations to further enhance NFPP performance.

High‐entropy doping is a strategy that produces a high‐entropy effect by introducing multiple doping elements into a material, showing promise in enhancing the electrochemical properties of NFPP. This approach increases the conformational entropy of the material, thereby improving its thermodynamic stability and kinetic properties [94, 95, 96]. Na_4_Fe_2.5_Mn_0.1_Mg_0.1_Co_0.1_Ni_0.1_Cu_0.1_(PO_4_)_2_(P_2_O_7_) cathode delivers a rate performance of 55 mAh g^−1^ at 10 A g^−1^ and an ultralong cycling lifespan exceeding 18 000 cycles at 5 A g^−1^ (Figure 8f) [90]. The increased configurational entropy in HE‐NFPP results in an improved structure, with a minimal volume variation of only 1.83% (Figure 8g). Likewise, the high‐entropy doped Na_4_Fe_2.85_(Ni,Co,Mn,Cu,Mg)_0.03_(PO_4_)_2_(P_2_O_7_) cathode achieves 122 mAh g^−1^ at 0.1C and 85 mAh g^−1^ at 50C [97].

Beyond high entropy, combining high entropy with an iron‐defect strategy can further enhance the electrochemical performance of NFPP. Na_4_Fe_2.65_(Ni,Cr,Mg,Co,Mn)_0.027_(PO_4_)_2_P_2_O_7_ was engineered with Fe defects to reduce impurity phase formation. This design achieves a capacity of 57 mAh g^−1^ at 100C and retains 80% of its capacity after 4000 cycles at 20C [98]. Another study developed a Na_4_Fe_2.61_(Ni,Co,Mn,Cu,Zn,Mg)_0.05_(PO_4_)_2_P_2_O_7_ cathode that combines high entropy doping with Fe vacancy engineering. By providing additional active sites and enhancing the stability of the crystal structure, this approach achieves a discharge capacity of 101.9 mAh g^−1^ and maintains 74.3% capacity retention after 15 000 cycles [99]. Combining high entropy with iron‐defect strategies can effectively enhance the electrochemical performance of NFPP, paving the way for more efficient and durable energy storage solutions.

While doping can significantly enhance various material properties, it also presents several potential challenges that are critical concerns in current research. The selection of doping elements offers substantial potential for expansion, especially for bi‐ionic doping, which remains relatively unexplored. For elements where doping has been implemented, the precision of doping levels often requires improvement, necessitating more accurate control of the ratios to achieve optimal performance. Additionally, doping can complicate material synthesis, especially in NFPP systems sensitive to impurities. There is a lack of in‐depth understanding of the specific mechanisms by which doping elements affect NFPP materials. Future research should focus on elucidating these mechanisms through theoretical calculations and experimental studies.

A comparative analysis of different modification strategies reveals their distinct roles in improving NFPP performance (Table 2). Carbon coating and conductive compositing are most effective in enhancing electronic conductivity, thereby significantly improving rate capability. Doping strategies primarily optimize the crystal structure and Na^+^ diffusion pathways, contributing to improved cycling stability and moderate rate performance. In contrast, impurity engineering and heterostructure design can introduce additional electrochemical activity or interfacial effects, in some cases leading to higher specific capacity. However, these strategies also present trade‐offs. Carbon‐based modifications may reduce tap density, while excessive doping can induce structural distortion. Impurity‐related approaches require precise control to avoid performance degradation. Both doping and impurity engineering are effective strategies for enhancing the electrochemical performance of NFPP; however, they involve distinct mechanisms and trade‐offs. Doping typically modifies the intrinsic electronic structure and ion diffusion pathways by introducing foreign elements into the lattice, leading to improved conductivity, structural stability, and redox activity. Nevertheless, excessive or inappropriate doping may introduce lattice distortion or increase synthesis complexity and cost. In contrast, impurity engineering focuses on controlling, suppressing, or even utilizing secondary phases (e.g., NaFePO_4_ or Na_2_FeP_2_O_7_) to enhance overall performance. While impurity suppression can improve phase purity and stability, impurity transformation strategies (e.g., heterostructure design) can sometimes deliver capacities beyond the theoretical limit of pure NFPP. However, controlling impurity phases precisely remains challenging, particularly in large‐scale synthesis. Therefore, the choice between doping and impurity engineering depends on the targeted performance metrics and application scenarios. In practice, combining these strategies may offer synergistic effects and represents a promising direction for future research. Overall, no single strategy is universally optimal; instead, combining multiple approaches often leads to synergistic improvements in conductivity, kinetics, and stability.

**TABLE 2:** Comparison of the electrochemical performances of NFPP cathodes in SIBs modified by different strategies.

Composite	Preparation method	Specific capacity, mAh g^−1^	Cycle number	Capacity retention	Ref
Mesoporous sponge‐like Na_4_Fe_3_(PO_4_)_2_(P_2_O_7_)@C@rGO	Solid‐state method	111.7@0.05C	30 000@20C	86.70%	[100]
Na_4_Fe_2.5_Mn_0.1_Mg_0.1_Co_0.1_Ni_0.1_Cu_0.1_(PO_4_)_2_(P_2_O_7_)@C	Sol–gel method	108.9@0.02A g^−1^	18 000@5A g^−1^	70.50%	[90]
Na_4_Fe_2.61_(Ni,Co,Mn,Cu,Zn,Mg)_0.05_(PO_4_)_2_(P_2_O_7_)@C	Sol–gel method	129.5@0.01A g^−1^	15 000@5A g^−1^	74.2%	[99]
Na_3.9_Fe_2.6_V_0.1_Mn_0.1_Cu_0.1_Mg_0.1_(PO_4_)_2_(P_2_O_7_)@C	Sol–gel method	122.3@0.1C	14 000@50C	78.58%	[95]
Nonstoichiometric Na_3.4_Fe_2.4_(PO_4_)_1.4_(P_2_O_7_)@C	/	110.8@0.1C	14 000@20C	83.10%	[61]
Na_4_Fe_3_(PO_4_)_2_(P_2_O_7_)@C‐Mg5%	/	104@0.05A g^−1^	14 000@5A g^−1^	80.80%	
Na_4.5_Fe_3.5_(PO_4_)_2.5_(P_2_O_7_)@C	Sol–gel method	130.5@0.1C	11 000@20C	88%	[49]
Na_3.94_Fe_2.94_V_0.06_(PO_4_)_2_(P_2_O_7_)@C	Spray‐drying method	126.4@0.1C	10 000@20C	81.65%	[66]
Micro porous Na_3.9_Fe_2.9_Al_0.1_(PO_4_)_2_(P_2_O_7_)@C	Ball milling and freeze‐drying method	115.2@0.1C	10 000@50C	85.10%	[80]
Na_4_Fe_2.85_Mn_0.15_(PO_4_)_2_(P_2_O_7_)@C	Solid‐state method	111.6@0.1C	10 000@50C	88.10%	[47]
Pure‐phase Na_4_Fe_2.91_(PO_4_)_2_(P_2_O_7_)@C	Spray‐drying method	110.9@0.2C	10 000@10C	Without decay	[25]
Hollow spherical Na_4_Fe_3_(PO_4_)_2_(P_2_O_7_)@C	Spray‐drying method	86.5@1C	10 000@10C	89.70%	[53]
Na_4_Fe_3_(PO_4_)_2_(P_2_O_7_)/Na_2_VTi(PO_4_)_3_@C	Spray‐drying method	155.3@0.02A g^−1^	2500@1A g^−1^	82.90%	[75]
Na_4_Co_0.5_Mn_0.5_Fe_2_(PO_4_)_2_(P_2_O_7_)@C	Solid‐state method	139@0.1C	2000@10C	65.20%	[91]
Na_4_Fe_3_(PO_4_)_2_(P_2_O_7_)@NaFePO_4_@C	Sol–gel fabrication method	136@0.1C	3000@10C	Without decay	[74]
Na_4_Fe_3_(PO_4_)_2_(P_2_O_7_)@C nanoparticles in carbon nanoribbons	Electrospinning method	128.6@0.1C	5000@50C	72%	[59]
Na_4_Fe_3_(PO_4_)_2_(P_2_O_7_)@C nanospheres	Template method	128.5@0.2C	4000@10C	63.50%	[46]
Na_4_Fe_2.82_Ni_0.18_(PO_4_)_2_(P_2_O_7_)@C‐N	Solid‐state method	128.4@0.2C	3000@10C	83%	[67]
Na_4_Fe_2.94_Al_0.04_(PO_4_)_2_(P_2_O_7_)@C	Solid‐state method	128.1@0.2C	3000@10C	83.70%	[62]
Na_4_Fe_3_(PO_4_)_2_(P_2_O_7_)@rGO microspheres	Spray‐drying method	128@0.1C	6000@10C	62.30%	[48]
Hollow Core‐Shelled Na_4_Fe_2.4_Ni_0.6_(PO_4_)_2_(P_2_O_7_)@C	Spray‐drying method	120.3@0.5C	5000@20C	72.96%	[79]
Na_4_Fe_2.98_Ti_0.01_(PO_4_)_2_(P_2_O_7_)@C	Spray‐drying method	112.5@0.1C	5000@10C	95.88%	[81]
Na_3_Fe_2_(PO_4_)(P_2_O_7_) (NFPP)@rGO	Spray‐drying method	105.5@0.2C	8000@20C	72.40%	[52]
Na_4_Fe_2.95_(NiCoMnMgZn)_0.01_(PO_4_)_2_(P_2_O_7_)@C	Spray‐drying method	105.3@0.1C	7000@10C	74.20%	[94]
Na_4_Fe_3_(PO_4_)_1.9_(SiO_4_)_0.1_(P_2_O_7_)@C	Solid‐state method	89.9@0.5C	7000@10C	79.40%	[92]

### 4.3 Surface Modification

Surface modification, particularly carbon modification, is a crucial method for enhancing the electrochemical performance of NFPP. Carbon modification is favored due to its affordability, high conductivity, and chemical stability. There are two primary approaches to achieving carbon coating: One involves introducing a carbon source during the synthesis process to form an in situ carbon coating on the NFPP surface via thermal decomposition. The other approach is to directly composite NFPP with conductive carbon materials to enhance its conductivity. These methods effectively optimize the performance of NFPP.

Various carbon sources have been explored to prepare in situ carbon coatings, including glucose, anthracite, folic acid, phytic acid (PA), citric acid, and organophosphonic acid in NFPP. These carbon sources can form a uniform carbon layer on the surface of NFPP during heat treatment, improving the conductivity and electrochemical properties of the material. By directly coating the surface of the material, the interfacial contact between the cathode material and the electrode can be improved, facilitating rapid electron transfer. This improvement in interfacial contact is crucial for enhancing the overall performance of the battery.

**FIGURE 9:**
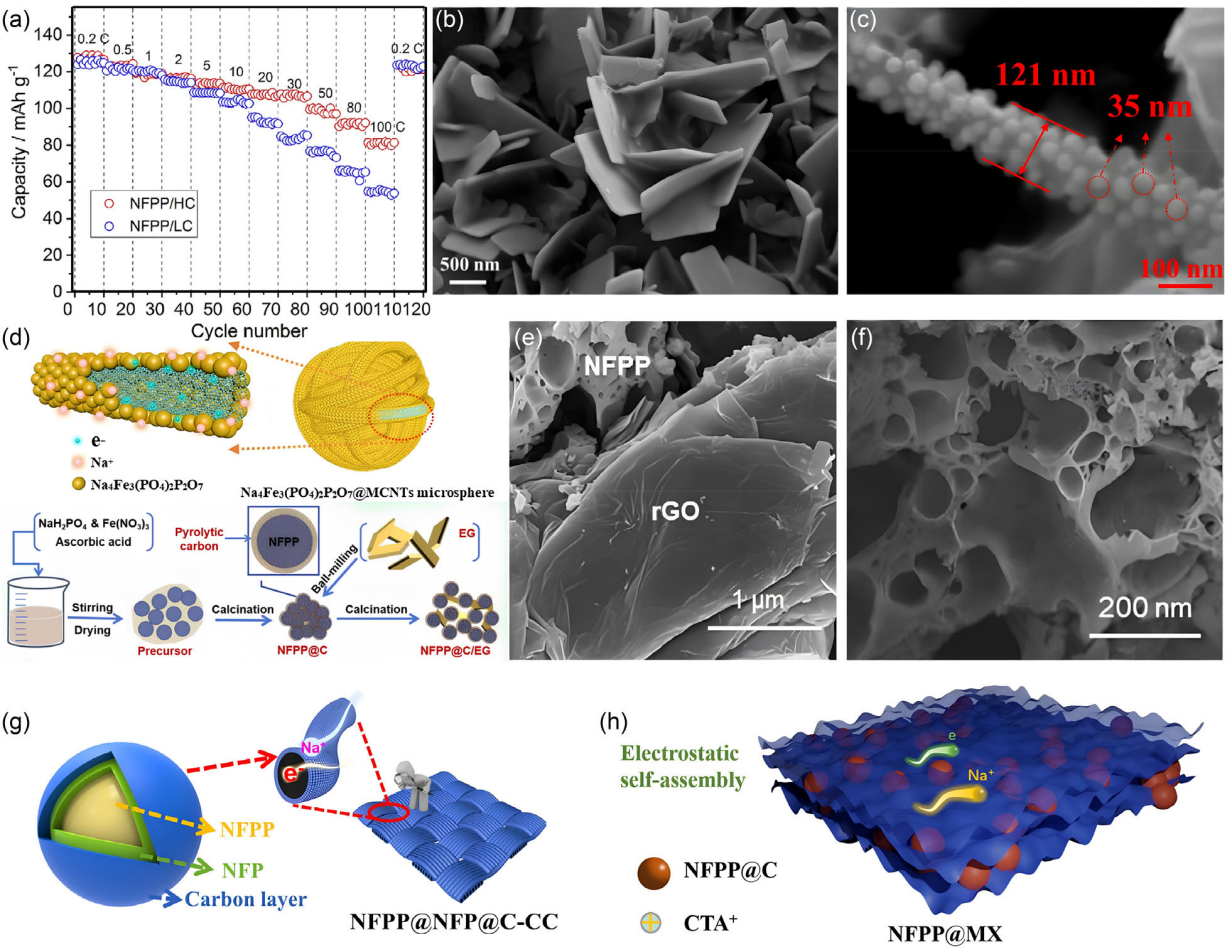
(a) Discharge capacities of NFPP/HC and NFPP/LC at various discharge current rates [46]. Reproduced with permission [48]. Copyright 2019, Elsevier. (b) SEM images of nanoflakes [101]. Reproduced with permission [102]. Copyright 2022, Elsevier. (c) SEM images of NFPP@MCNTs [103]. Reproduced with permission [104]. Copyright 2020, Elsevier. (d) Illustration of the synthesis process of NFPP@MCNTs [103]. Reproduced with permission [104]. Copyright 2020, Elsevier. (e,f) SEM images of mesoporous sponge‐like NFPP@C@rGO [100]. Reproduced with permission [105]. Copyright 2023, Elsevier. (g) Schematic illustration of the preparation process of NFPP@NFP@C‐CC flexible electrode [74]. Reproduced with permission [78]. Copyright 2019, Elsevier. (h) Schematic illustration of the synthesis of NFPP@MX [105]. Reproduced with permission [106]. Copyright 2024, MDPI.

Interestingly, NFPP can achieve a capacity close to its theoretical maximum solely with an appropriate carbon coating, without additional modification methods. The synthesized NFPP/C nanospheres achieve a high discharge capacity of 128.5 mAh g^−1^ at 0.2C, with a capacity retention of 63.5% after 4000 cycles at 10C [46]. Notably, a high reversible capacity of 79 mAh g^−1^ is demonstrated at an ultrahigh current rate of 100C (Figure 9a). The nanospheres (~30 nm) and carbon coating layers (~3 nm) create efficient conductive channels for electrons, resulting in excellent performances.

Introducing heteroatoms into the in situ carbon coating can further optimize the electrochemical performance of NFPP. Generally, carbon materials contain numerous defects, seriously affecting their comprehensive performance. Nitrogen doping can effectively reduce these defects, improving their stability and conductivity. A hierarchical nitrogen–carbon‐coated NFPP nanoflake (Figure 9b) was prepared, in which oleic acid is the surfactant and carbon source [101]. These NFPP nanoflake composites operate over a wide temperature range from −20°C to 50°C, providing a capacity of 129 mAh g^−1^ at 0.1C, retaining 81% capacity at 10C after 1500 cycles at 25°C. A low‐cost and simple nitrogen doping method is expected to fully exploit the potential of coating materials and enhance the performance of NFPP, which requires more attention.

Another simple but effective strategy involves a direct dispersion of carbon nanotubes in NFPP precursors and high‐temperature sintering. Carbon nanotubes can significantly enhance the electron conductivity and stability of NFPP, which enhances its electrochemical performance. NFPP nanospheres were developed and grown on multiwalled carbon nanotubes (MCNTs) (Figure 9c,d) [103]. The NFPP@MCNT composite exhibits a capacity of 115.7 mAh g^−1^ at 0.1C and retains 95% of its capacity after 1200 cycles at 2C and the rate capability of 62.8 mAh g^−1^ can be seen even at 20C. Another study also developed NFPP@CNT‐1%, which delivers a capacity of 103.9 mAh g^−1^ at 0.1C and retains 99.9% of its capacity after 1000 cycles at 5C [102]. These advancements underscore the potential of carbon nanotubes in boosting the electrochemical performance of NFPP materials for better energy storage.

The synthesized graphene Na_3_Fe_2_(PO_4_)(P_2_O_7_) cathode demonstrated an ultralong cycle life, retaining 72.4% of its capacity after 8000 cycles at 20C, and exhibited outstanding rate capability, achieving 42.4 mAh g^−1^ at 100C, largely due to graphene's ability to provide a robust conductive network that facilitates rapid electron transfer [52]. In another study, 3D graphene‐decorated Na_4_Fe_3_(PO_4_)_2_(P_2_O_7_) microspheres were prepared using spray‐drying method. This composite achieved a capacity of 128 mAh g^−1^ at 0.1C and maintained 62.3% capacity retention over 6000 cycles at 10C [48]. Numerous studies have highlighted the effectiveness of graphene in surface modification, primarily due to its ability to significantly enhance electronic conductivity and alleviate structural stress in NFPP, thereby boosting overall performance during charging and discharging cycles.

The dual‐carbon modification enhances the performance of NFPP by improving electronic conductivity and facilitating Na^+^ ion diffusion. An NFPP composite, integrated with in situ carbon and expanded graphite, achieved a rate capability of approximately 80.5 mAh g^−1^ at 50C and cycling stability with 89.85% capacity retention over 11 000 cycles [104]. Another mesoporous NFPP structure with a carbon layer and graphene nanosheets resulted in 86.7% capacity retention at 20C for over 30 000 cycles and excellent thermal stability at 60°C (Figure 9e,f) [100]. Furthermore, NFPP@C/HG, synthesized through a scalable ball milling strategy and modified with 3D holely graphene, delivers a capacity of 118 mAh g^−1^ at 0.2C and maintains 53 mAh g^−1^ even at 100C [106]. Additionally, NFPP@AC/rGO exhibits 78 mAh g^−1^ at 20C and 30°C and 42 mAh g^−1^ at 20C and 15°C [107]. These dual‐carbon modification strategies significantly enhance the electrochemical performance of NFPP, particularly in long‐term stability, highlighting their potential for practical applications.

Other strategies for surface modification were employed in the fabrication of the NFPP cathode material. Metal‐phosphate capping can improve electronic conductivity and provide more active sites [74]. The Na_4_Fe_3_(PO_4_)_2_(P_2_O_7_)@NaFePO_4_@C was prepared using a sol–gel fabrication strategy (Figure 9g). This composite exhibits a discharge capacity of this composite is 136 mAh g^−1^ at 0.1C, with no capacity decay over 3000 cycles at 10C and an excellent rate capability of 68 mAh g^−1^ at 100C. Additionally, 3D network structures assisted by MXene represent another important modification method to enhance the electrochemical performance of NFPP. The Na_4_Fe_3_(PO_4_)_2_(P_2_O_7_)@C/Ti_3_C_2_T_*x*_ was synthesized through an electrostatic self‐assembly method (Figure 9h) [105]. By creating this cross‐linked structure, the interparticle contact area is increased, and electrical conductivity is enhanced, leading to a discharge capacity of 106.1 mAh g^−1^ at 0.2C and a rate capability of 60.4 mAh g^−1^ at 10C.

The advancement of NFPP cathode materials necessitates precise control over the thickness and uniformity of the carbon cladding layer on the active material's surface. These properties of the carbon layer directly influence the Na^+^ migration efficiency between the particles and the electrolyte, affecting the charging and discharging performance of the battery. An inhomogeneous or excessively thick carbon layer may hinder ion transport, whereas a uniform and moderately thick carbon layer optimizes the ion and electron transport paths and improves the electrochemical activity of the material.

## 5 From Na_4_Fe_3_(PO_4_)_2_(P_2_O_7_) Material to Practical Application

We have reviewed the properties, synthesis, and modification strategies of NFPP materials and evaluated their electrochemical performance in half‐cells. Nonetheless, data from half‐cell tests may not apply to sodium‐ion full battery (SIFC) systems. To fully assess NFPP cathodes, further discussion on electrolytes and anodes is needed. The commercialization of NFPP is advancing rapidly, showing strong market potential and innovation. Challenges such as compaction density, presodium strategies, and cost issues will be mentioned.

### 5.1 Full Cell

The commercial use of NFPP materials for SIB applications is progressing, with potential applications in multiple battery forms being investigated. Beyond half‐cells, NFPP is adapted for button, pouch, and cylindrical cells. Selecting appropriate anode materials and electrolytes with adequate initial Coulomb efficiency and operating potential is essential for the full cell.

**FIGURE 10:**
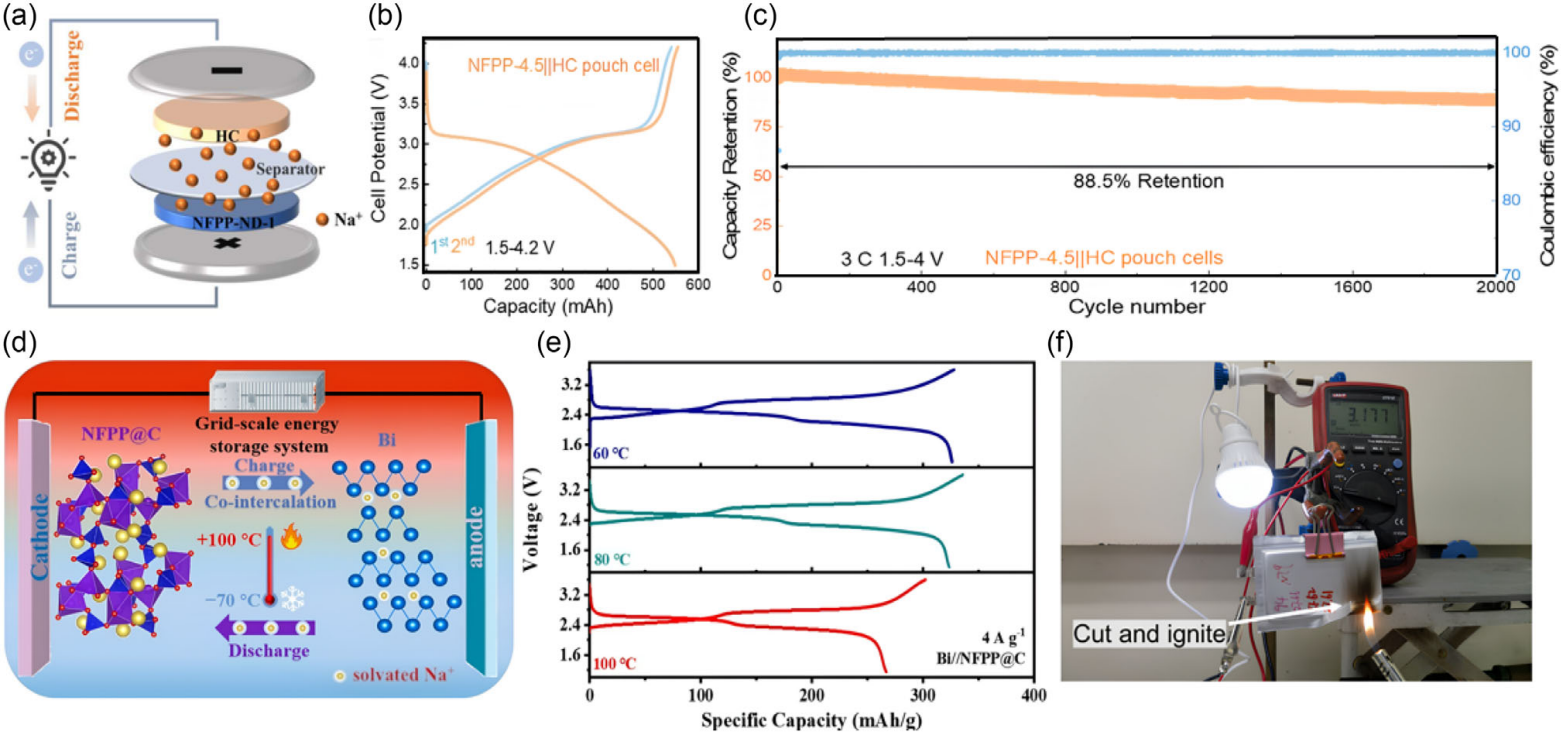
(a) The button cell schematic [69]. Reproduced with permission [78]. Copyright 2025, Royal Society of Chemistry. (b,c) Initial charge/discharge profile and cycling performance at 3C for NFPP‐4.5 pouch cells [49]. Reproduced with permission [51]. Copyright 2024, American Chemical Society. (d) Cointercalation process of solvated Na^+^ ions [108]. Reproduced with permission [109]. Copyright 2022, Wiley. (e) Galvanostatic charge/discharge curves of Bi//NFPP@C battery at 60°C, 80°C, and 100°C [108]. Reproduced with permission [109]. Copyright 2022, Wiley. (f) Photo of the fully charged HC//NFPP pouch cells after package clipping and under a flame [110]. Reproduced with permission [111]. Copyright 2024, American Chemical Society.

Prototypes of SIFC batteries include button, pouch, and cylindrical cells, each differing in internal structure and encapsulation. As shown in Figure 10a, button cells are the most common form in research, aiding in evaluating the performance of the NFPP cathode. Compared to button cells, pouch cells are gaining attention because of their high safety, low internal resistance, and design flexibility. A pouch cell was prepared with 2 kg of Na_4_Fe_2.91_(PO_4_)_2_(P_2_O_7_) to assess its electrochemical performance, maintaining a capacity of 85 mAh g^−1^ after 1000 cycles at 1C with no observable degradation [25]. Another study prepared 2 kg of Na_4.5_Fe_3.5_(PO_4_)_2.5_(P_2_O_7_) to examine practical applications. The capacity of the small pouch can be reversed at approximately 550 mAh at 0.1C between 1.5 and 4.2 V, retaining 88% capacity even after 2000 cycles (Figure 10b,c).

Cylindrical cells can exhibit higher energy density, more consistent product quality, and reduced cost. Notably, flatulence issues remain a challenge for SIBs. Compared to square and pouch cells, cylindrical cells have a rigid structure that enhances resistance to swelling. Their flexibility allows cylindrical cells to form various modules, facilitating the expansion of SIBs across multiple applications and supporting commercialization. Although data on cylindrical cells with NFPP cathodes is lacking, these cells hold strong potential for future commercialization.

Compatibility between the anode and cathode is crucial for achieving excellent electrochemical performance in batteries. Hard carbon (HC) is frequently utilized as an anode material in full cells because of its high reversible capacity and excellent cycling stability. The NFPP/HC anode is highly effective in SIBs, offering a power density of 24.1 kW kg^−1^ and an energy density of 146.6 Wh kg^−1^, resulting in high power and energy density. Additionally, compounds like NaTi_2_(PO_4_)_3_ (NTP) have been investigated for use with NFPP cathode materials. A recent study reported the HE‐NFPP//HC battery has a specific capacity of 82.8 mAh g^−1^ at 0.2 A g^−1^, while the HE‐NFPP//NTP cell exhibits a capacity of 85.7 mAh g^−1^ at 50 mA g^−1^ current [90]. After 200 cycles, the HE‐NFPP/NTP cell remains at 99.2% of its capacity, highlighting the cycling stability advantages of NTP as an anode material. Another study used a bismuth (Bi) anode in a Bi//NFPP@C battery [108]. At −70°C, the Bi//NFPP battery retains 70.19% of its capacity at room temperature and performs well at temperatures up to 100°C, benefiting from its superior electrolyte boiling point (Figure 10d,e).

Selecting the appropriate electrolyte is essential for SIB performance, as it requires high ionic conductivity to ensure efficient sodium ions transport between electrodes. Various electrolyte formulations have been studied for NFPP cathode materials, including NaClO_4_ dissolved in EC/DEC carbonate electrolyte [53], NaPF_6_ in EC/PC (with FEC additive) [66], PD, PTD [112], and NaPF_6_/DEGDME. A cell with EC/PC/1M NaClO_4_ and an NFPP cathode exhibits a highly reversible capacity of 122 mAh g^−1^ [112]. After 100 cycles, the discharge capacity remains nearly constant with a high Coulombic efficiency of 99%. Replacing NaPF6/DEGDME with NaPF_6_/PC 5% FEC for the NFPP‐Mg5% cathode results in a higher operating voltage and improved discharge plateau. Despite the initial performance, cells with NaPF_6_/DEGDME electrolyte face decreasing discharge capacity after 100 cycles. Optimizing the electrolyte is crucial for enhancing the electrochemical performance of NFPP full cells, requiring further attention to address commercial use challenges. An electrolyte (NaClO_4_:TMP:TFE*P* = 1:3:2, by mol, 1.22 M) that is nonflammable, cost‐effective, and low salt concentration was developed [110]. The Ah‐level HC/NFPP pouch cell achieves CE of over 99.9% and retains 84.5% capacity over 2000 cycles, showing excellent performance across temperatures from −20°C to 60°C. This pouch cell maintains a steady voltage output despite packaging clipping and flame exposure, indicating its safety (Figure 10f).

### 5.2 Commercialization

**FIGURE 11:**
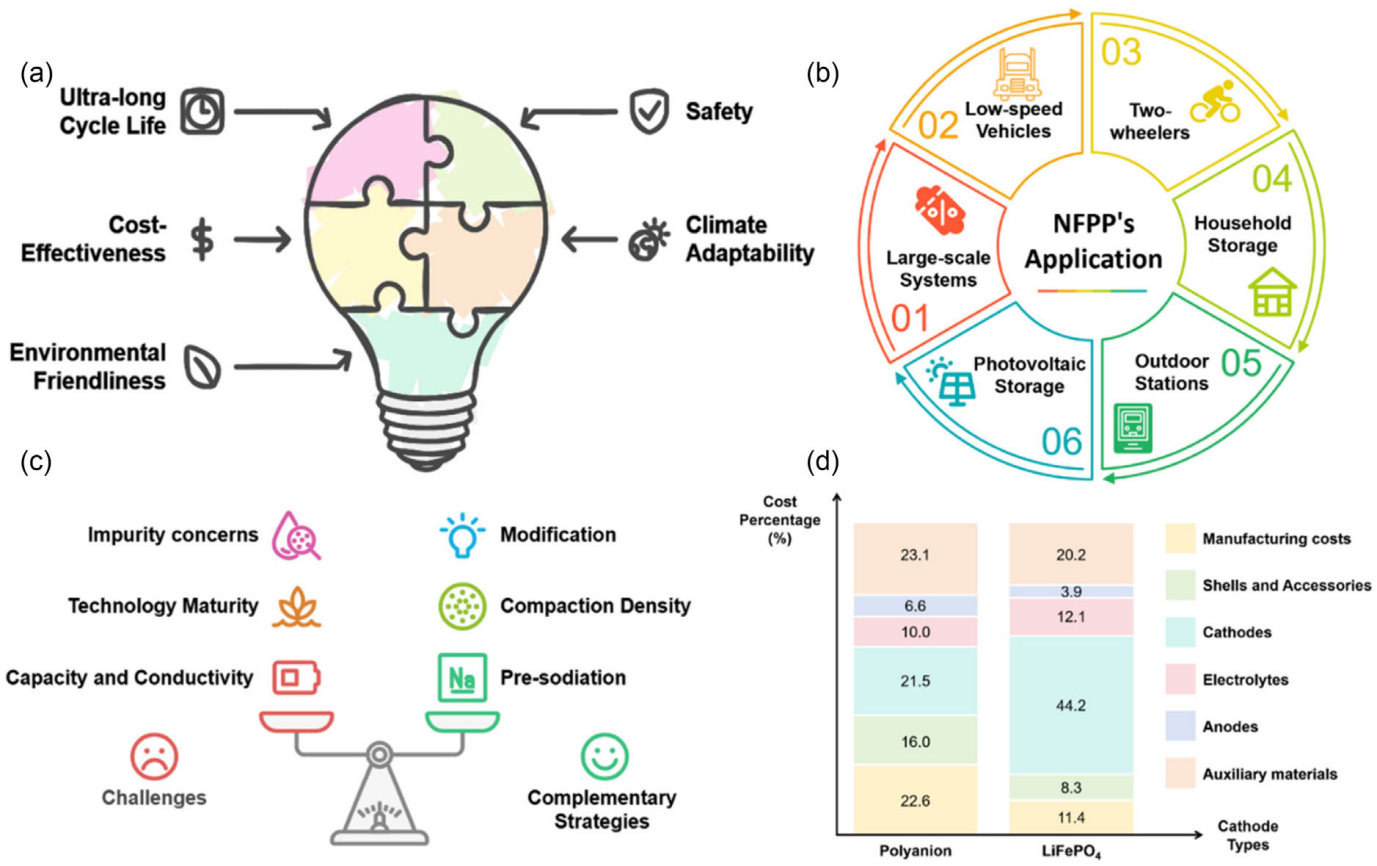
(a) The advantages of NFPP for commercialization. (b) NFPP's application for commercialization. (c) The challenges and complementary strategies of NFPP. (d) The cost percentage analysis for SIBs with polyanion cathodes and LiFePO_4_ cells.

Against the background of global energy structure transformation and upgrading, the demand for energy storage technologies is increasing. These energy storage technologies prioritize cycle life, safety, cost‐effectiveness, and environmental friendliness (Figure 11a). These technologies are widely applied in fields such as large‐scale energy storage systems, low‐speed vehicles, two‐wheelers, household storage, outdoor base station energy storage, and photovoltaic energy storage (Figure 11b). Among various competing cathode materials for SIBs, NFPP stands out due to its ultralong cycle life, climate adaptability, and high safety in energy storage systems. Additionally, NFPP is cost‐effective and environmentally friendly as it lacks toxic or expensive elements like vanadium (V), cobalt (Co), nickel (Ni), chromium (Cr), and fluorine (F). With these remarkable properties, NFPP is an attractive candidate for energy storage applications and has become a prominent focus of research and commercialization in academia and industry.

The industrial progress of NFPP cathode materials in SIBs has shown a positive trend and is currently in a critical stage of its industrialization process. This period has seen a steady increase in the technological maturity of NFPP, with the industry recognizing its potential in SIBs. The latest information demonstrates that many companies are actively promoting the development and industrialization of NFPP materials. Although NFPP is less mature than some traditional cathode materials and still facing challenges, its unique advantages and complementary strategies strongly support its potential for large‐scale commercialization (Figure 11c). The pace of industrialization of NFPP materials is expected to accelerate significantly as work progresses and technical difficulties are systematically overcome. This will boost the development of SIB technology and contribute to the development of the entire energy storage industry.

#### 5.2.1 A Comparative Examination of Potential Competitive Cathodes

Among the promising cathode materials for SIBs, which compete as potential alternatives, there is no clear consensus on which material offers superior performance. To fully understand the advantages and limitations of NFPP cathode materials, it is necessary to compare other polyanion cathode materials with commercialization prospects. Recent studies indicate that the most promising polyanion cathode materials include phosphate, pyrophosphate, and sulfate.

Lead‐acid and lithium‐iron phosphate batteries are significant competitors to NFPP, influencing each other's commercialization. As demand for lithium batteries rises, SIBs are primarily used as complementarity and effective substitutes. Nonetheless, SIBs, particularly those with iron‐based NFPP cathodes, are more cost‐effective than lithium‐iron‐phosphate cathodes. Additionally, LIBs cannot match the all‐weather resilience of SIBs. For instance, the Bi/NFPP@C battery, whose capacity maintains 70.19% of its room temperature even at −70°C, still excels at temperatures up to 100°C. Compared to lead‐acid batteries, NFPP offers advantages in energy density, life cycle, and cost, potentially leading to the gradual phase‐out of lead‐acid batteries.

Since their introduction, sodium vanadium phosphate cathodes have garnered significant attention for their excellent electrochemical properties. Additionally, cathode materials with commercial potential, such as Na_3_V_2_(PO_4_)_2_F_3_ (NVPF) and Na_3_(VO)_2_(PO_4_)_2_F (NVOPF), have been developed and applied in practical applications. The energy density of NFPP cathode materials is lower than that of vanadium‐based due to their low redox potential. The cost‐effectiveness and environment‐friendly properties of NFPP make it more competitive than vanadium‐based phosphates, especially in applications that do not require high energy density. NFPP materials are expected to gain popularity in low energy density applications, while V‐based materials will remain important for high energy density needs.

Sodium iron sulfate is increasingly becoming a focus of research and application among sulfate cathode materials due to its unique advantages. Both sodium iron sulfate and sodium iron phosphate pyrophosphate offer benefits such as lower cost, medium energy density, and environmental friendliness. Compared to sodium iron phosphate pyrophosphate, sodium iron sulfate faces a technical challenge in its water absorption. At high temperatures, this problem worsens, leading to low‐capacity and iron ion leaching, harming the stability of the electrode–electrolyte interface. Despite this, the relatively low cost of sodium iron sulfate makes it suitable for small power applications, such as two‐wheeled vehicles, while NFPP may be more appropriate for energy storage needs. Notably, there is no consensus on whether sodium iron sulfate or sodium iron phosphate pyrophosphate is superior in commercial applications. NFPP and Na_2_Fe_2_(SO_4_)_3_ are expected to follow the commercially successful LiFePO_4_ preparation method. As technology advances, both materials are expected to achieve their commercialization potential in appropriate applications.

Compared with other cathode materials for SIBs, NFPP exhibits a unique balance of safety, cost, and cycling stability. Layered oxide cathodes generally offer higher energy density and better electronic conductivity; however, they suffer from structural instability, air sensitivity, and safety concerns, especially under high voltage and long‐term cycling conditions. Prussian blue analogs, on the other hand, are attractive due to their low cost and facile synthesis, but their performance is often limited by structural defects, lattice water, and relatively low volumetric energy density. In contrast, NFPP, as a polyanion‐type cathode material, benefits from a robust 3D framework, high thermal stability, and excellent cycling durability, making it particularly suitable for large‐scale energy storage applications where safety and lifespan are critical. However, its relatively low electronic conductivity and the presence of impurities remain key challenges. NFPP occupies a distinctive position among sodium‐ion cathodes, offering a compelling trade‐off between performance, safety, and cost, especially for grid‐scale energy storage systems.

#### 5.2.2 Compaction Density

Compaction density is a critical parameter for the commercialization of NFPP, significantly affecting its electrochemical performance. A higher compaction density allows for more active material, thus increasing the energy density of SIBs. Nonetheless, excessively high compaction density can reduce specific capacity and cycling stability. Therefore, understanding and controlling compaction density is essential for optimal performance. Reports indicate that the compaction density of NFPP in commercial products has reached 2.2 g/cm^3^, with the particles remaining intact and processable. Another study reported a sample of NFPP, with a compacted density of 1.92 g/cm^3^ [25]. Surface coating and morphology control can enhance the electrochemical properties of phosphate‐based polyanionic compounds [8]. Nonetheless, these strategies ultimately impact the performance of actual SIBs by reducing the electrode capacity or compact density, compromising the energy and volume density of the final product. To enhance the performance of NFPP full cells without affecting compaction density, greater attention should be given to modification strategies such as electrolyte optimization and element doping.

In addition to electrochemical performance, practical deployment of NFPP cathodes requires careful consideration of tap density and slurry processability. Phosphate‐based polyanion materials, including NFPP, typically rely on carbon coating or carbon compositing to improve electronic conductivity; however, this often leads to reduced tap density due to the low intrinsic density and porous nature of carbon. A low tap density directly limits the achievable volumetric energy density, which is a critical parameter for commercial SIBs. Moreover, synthesis routes that produce highly porous or nanosized particles, while beneficial for rate capability, may further decrease packing efficiency and increase electrode tortuosity. From a processing standpoint, particle morphology, size distribution, and surface chemistry strongly influence slurry rheology and electrode fabrication. For instance, excessive surface area and irregular particle shapes can increase binder demand and slurry viscosity, leading to poor coatability and inhomogeneous electrode films. Conversely, spherical secondary particles (e.g., obtained via spray drying) with controlled size distribution can improve flowability, packing density, and electrode uniformity. Therefore, future development of NFPP materials should aim to balance nanoscale electrochemical advantages with microscale engineering strategies, such as secondary particle design and optimized carbon distribution, to simultaneously achieve high gravimetric and volumetric performance while maintaining good manufacturability.

#### 5.2.3 Presodiation Strategy

In the practical application of NFPP full batteries, the presodiation strategy is crucial for enhancing the electrochemical performance of SIFCs, as it compensates for the initial loss of sodium ions and extends battery cycle life. Presodiation techniques include three main strategies: electrochemical presodiation process, chemical presodiation strategies, and the addition of additives to the cathode material. Electrochemical presodiation has advanced in laboratory settings for presodiation electrodes, but commercialization remains challenging due to complexity and performance limitations. Chemical presodiation is effective for sodium‐ion prestorage; however, their industrial viability is constrained by the limited reduction capacity of the solution and the time‐consuming treatment process.

To compare, cathode additives have been widely utilized in commercial LIBs, providing valuable insights into the presodiation strategies of NFPP. In a recent study, introducing commercial Na_2_C_2_O_4_ into the NFPP cathode increased the initial capacity from 111 to 140 mAh g^−1^ [109]. At a current density of 0.1C, the reversible specific capacity of the HC||NFPP increased from 83 to 103 mAh g^−1^, resulting in an energy density of 305 W h kg^−1^ at 0.2C. After 400 stable cycles, the capacity retention remains 81% at 1C. Cathode additives can directly enhance the energy density of SIB systems, offering significant potential for commercialization. Despite the importance of presodiation strategies for NFPP, relevant studies remain relatively limited. Therefore, developing cathode additives suitable for NFPP cathode materials with excellent stability in air and low cost is crucial for their commercialization process.

We believe that NFPP will become increasingly prominent, with more companies engaging in its commercialization. While one material may eventually dominate, the likely trend is not the dominance or elimination of a single cathode material, but rather complementary roles and differentiated competition among various cathode materials. Each cathode material has unique strengths and limitations, and the most suitable choice should be based on a comprehensive assessment of performance needs and the specific application environment.

#### 5.2.4 Cost Analysis

The cost of batteries is heavily influenced by cathode materials, which is crucial in determining their commercialization scalability. The absence of costless cathode materials was a barrier to SIBs commercialization in the past. Nonetheless, NFPP material cathode has made SIBs commercialization feasible due to its affordability and adaptability to large‐scale production.

The cost of SIBs is influenced by various factors, including raw material costs, electrode electrochemical performance, and packaging expenses. For instance, the cathode accounts for 21.51% of the cost in SIBs with polyanion cathodes compared to 41.17% in LiFePO_4_ cells (Figure 10d) [8]. The use of cheaper materials is a key reason for the cost advantage in commercializing SIBs, especially for NFPP cathodes. Despite the low cost of its raw materials, NFPP incurs significantly higher expenses in manufacturing, packaging, and ancillary components due to its inferior volumetric capacity density relative to LIBs. Indeed, these combined costs for SIBs are nearly double those of LIBs (38.6% vs. 19.7%). The cathode cost contribution is also highly dependent on technological readiness, though ongoing advancements in the NFPP supply chain and fabrication processes promise continued cost reductions alongside scaling. Although current low Li_2_CO_3_ prices challenge SIB competitiveness, the inherent cost volatility of lithium reinforces NFPP as the superior long‐term solution for grid‐scale storage. Any substantial increase in lithium carbonate prices would therefore catalyze a decisive market shift toward NFPP.

#### 5.2.5 TRL Comparison

**FIGURE 12:**
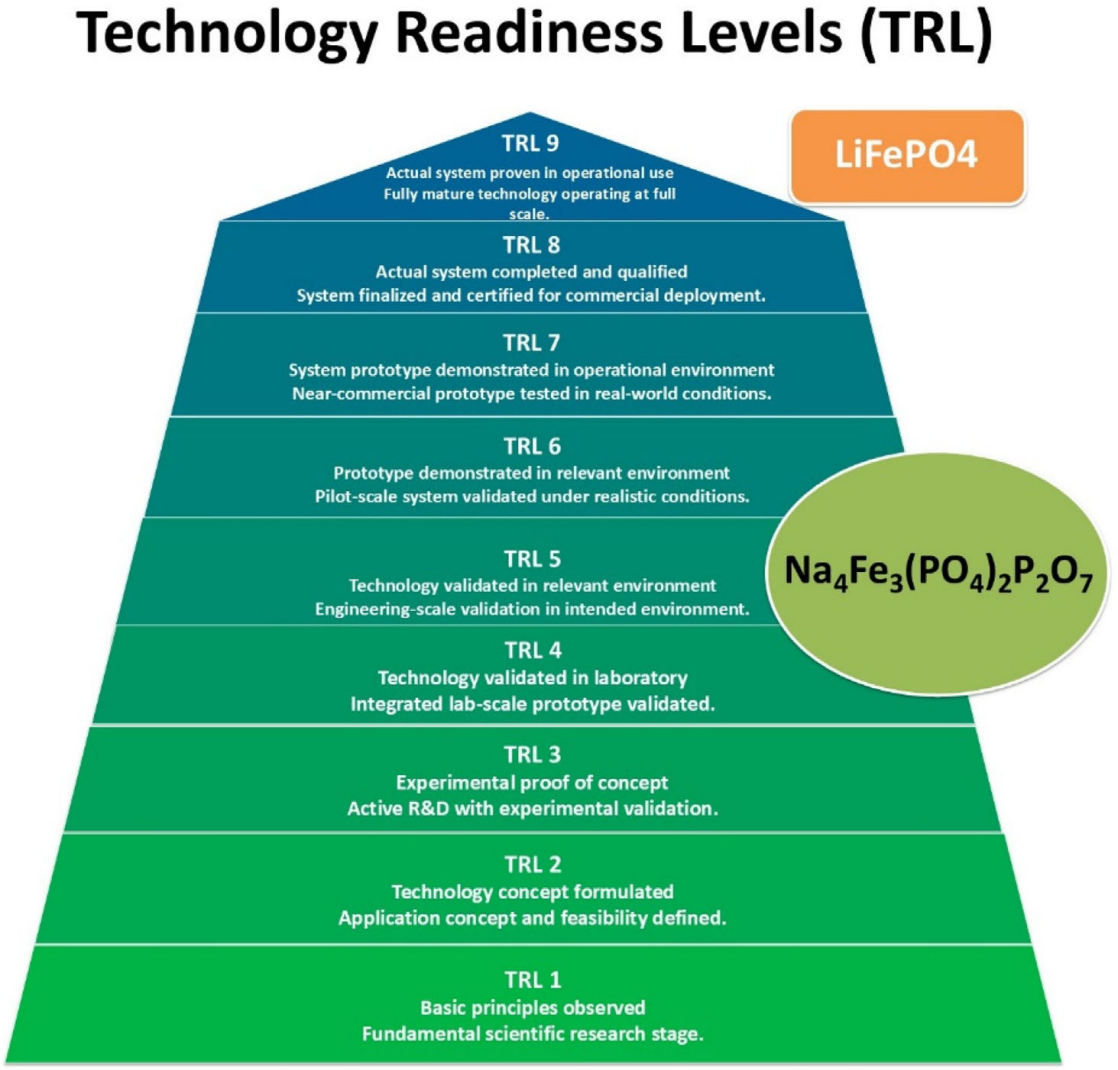
The comparison of TRL for LiFePO_4_ and Na_4_Fe_3_(PO_4_)_2_P_2_O_7_.

As illustrated in Figure 12a, LiFePO_4_ has reached Technology Readiness Level (TRL) 9, indicating full technological maturity from material design and scalable synthesis to electrode fabrication, cell integration, and commercial deployment. Its success stems not only from the intrinsic structural robustness of the olivine framework and superior thermal stability but also from long‐term engineering optimization, including carbon coating and particle downsizing strategies, standardized slurry and electrode processing, highly compatible pairing with graphite anodes, and the establishment of a complete and resilient supply chain. Consequently, LiFePO_4_ has achieved strong cross‐scale integration across materials, cells, battery systems, and end‐use scenarios, with validation at the GW–TWh scale in electric mobility and stationary storage applications, thereby substantially mitigating both technical and commercial risks.

In contrast, Na_4_Fe_3_(PO_4_)_2_P_2_O_7_ is currently positioned at TRL 4–6. This level reflects successful laboratory validation and prototype demonstration in relevant environments, yet it remains short of large‐scale commercial deployment. At the materials level, although its 3D open framework enables favorable Na^+^ diffusion kinetics and structural stability, further advances are required in enhancing electronic conductivity, achieving reproducible and scalable synthesis with controlled particle morphology, and ensuring stability under high‐voltage operation. At the electrode and full‐cell level, challenges persist in optimizing compatibility with HC anodes, stabilizing the electrolyte window, suppressing interfacial side reactions, and mitigating polarization under high mass loading. From an engineering perspective, industrial‐scale consistency, energy‐efficient production routes, comprehensive cost modeling, and long‐term cycling reliability under practical conditions remain to be systematically validated—key factors limiting its progression beyond TRL 6.

Importantly, the developmental trajectory of Na_4_Fe_3_(PO_4_)_2_P_2_O_7_ may draw instructive parallels from that of LiFePO_4_. The commercialization of LiFePO_4_ demonstrates that once a polyanionic cathode system achieves a balanced interplay among structural stability, safety, and cost, sustained advances in interface engineering and electrode architecture can enable rapid TRL acceleration. For Na_4_Fe_3_(PO_4_)_2_P_2_O_7_, the intrinsic advantages of earth‐abundant iron and sodium resources, combined with its favorable safety characteristics, align particularly well with the demands of large‐scale stationary energy storage, where cost efficiency, durability, and resource sustainability are prioritized over extreme energy density. In such grid‐oriented applications—including peak shaving and distributed storage—the material's technoeconomic profile is strategically compelling.

Therefore, the current TRL gap between Na_4_Fe_3_(PO_4_)_2_P_2_O_7_ and LiFePO_4_ does not fundamentally arise from insurmountable materials‐science limitations, but rather from the absence of fully established engineering frameworks and industrial ecosystems. With continued progress in standardized large‐scale synthesis, holistic full‐cell design, long‐term reliability assessment, and pilot‐scale demonstration projects, Na_4_Fe_3_(PO_4_)_2_P_2_O_7_ is well positioned to transition from laboratory validation to system‐level demonstration and early‐stage commercialization. Such advancement will be pivotal in determining whether this cost‐effective sodium‐ion cathode can replicate—or in specific stationary storage scenarios, even surpass—the mature development pathway exemplified by LiFePO_4_.

## 6 Summary and Outlooks

From a broader perspective, SIB cathodes can be broadly classified into layered oxides, polyanion‐type compounds, and Prussian blue analogs, each with distinct advantages and limitations. Among them, NFPP, as a representative polyanion‐type material, exhibits several intrinsic merits, including a robust 3D framework, high thermal stability, and excellent cycling durability. These features make NFPP particularly attractive for large‐scale energy storage applications, where safety, lifespan, and cost are prioritized over energy density.

NFPP with a NASICON structure has garnered increasing attention as a promising SIB cathode material due to its low cost and excellent electrochemical performance. This review summarizes the research progress on NFPP, focusing on its crystal structure, electrochemical properties, and synthesis methods. Additionally, it provides an in‐depth analysis of modification strategies aimed at enhancing NFPP's electronic conductivity for improved electrochemical performance. The review also examines the development of NFPP full cells and the current state of commercialization. However, NFPP also faces several challenges that hinder its practical deployment. Its intrinsically low electronic conductivity necessitates additional modification strategies, such as carbon coating or compositing, which may increase processing complexity. Moreover, impurity phase formation during synthesis remains difficult to control and can significantly influence electrochemical performance. Compared with layered oxides, NFPP generally exhibits lower energy density, while compared with Prussian blue analogs, its synthesis process is relatively more complex and less flexible. To address these gaps, the following three research priorities are proposed:

Green and cost‐effective synthetic approaches for impurity‐free, large‐scale production need further exploration to enable commercialization. Current synthesis techniques for NFPP material are inadequate to ensure the purity required for practical applications. Additionally, the challenge of fully utilizing or suppressing impurities to achieve high‐performance material remains unresolved and requires further research. By employing customized synthesis conditions, it is possible to investigate the mechanism behind the synergistic interaction of heterostructure.

Machine learning is expected to help predict nonstoichiometric pure phases of NFPP material and assist in doping processes. Traditionally, the electrochemical performance of new systems is verified through repeated experiments, which, although accurate, involve a lengthy trial‐and‐error process. In contrast, machine learning offers a more efficient approach to exploring multiple systems and can provide valuable insights for researchers. By combining DFT calculation and machine learning with various arithmetic, it is possible to predict the properties and performance of the new system rapidly.

A rational NFPP morphology and size design needs more investigation, which can favor electron and ion diffusion paths. Nanostructures can significantly enhance the initial Coulombic efficiency of SIBs by increasing surface area and reaction sites, facilitating ion diffusion, and optimizing the solid electrolyte interphase. Additionally, porous structures further aid by improving ion and electron transport and accommodating volume changes, enhancing overall stability and efficiency. Nonetheless, limited NFPP morphologies have been reported to date. The design of NFPP can be accelerated by taking advantage of the experience of LIBs and other polyanionic cathodes in controlling their morphology. To sum up, the industrialization of NFPP cathode materials is presently at TRL 4–6. Although laboratory validation and prototype testing are established, significant advances in technological maturity and market readiness are necessary. Despite existing hurdles, emerging practical applications will undoubtedly drive continued research momentum.

In summary, although significant progress has been made, the practical application of NFPP is still constrained by several key challenges, including low electronic conductivity, difficulty in impurity control, and limitations in scalable synthesis. Future efforts should focus on developing strategies that simultaneously address these issues, particularly through scalable processing techniques combined with effective structural and compositional optimization. A clear understanding of these challenges will be crucial for advancing NFPP toward real‐world energy storage applications.

Looking forward, future research should focus on developing scalable and cost‐effective synthesis routes, achieving precise control over phase purity and microstructure, and designing synergistic modification strategies to enhance electronic conductivity and sodium‐ion diffusion kinetics. With continued progress, NFPP is expected to play a significant role in next‐generation SIBs, particularly for grid‐scale energy storage and other applications requiring high safety and long service life. NFPP represents a promising candidate for safe, low‐cost, and durable SIBs, particularly in stationary energy storage systems where long‐term reliability is critical. In addition, there are several key directions for the development of NFPP cathodes. First, advanced characterization techniques, particularly in situ/operando methods, are needed to better understand phase evolution and sodium‐ion storage mechanisms during cycling. Second, interface engineering, including surface coating and electrode/electrolyte optimization, is critical for improving interfacial stability and long‐term performance. Third, the development of scalable and cost‐effective synthesis routes with precise control over phase purity and microstructure remains essential for industrial application. At the same time, several challenges must be addressed for practical commercialization, including intrinsically low electronic conductivity, difficulty in impurity control, and the trade‐off between performance and processing cost. Addressing these issues through integrated material design and engineering strategies will be crucial for advancing NFPP toward large‐scale energy storage applications.

## Conflicts of Interest

The authors declare no conflicts of interest.

## Data Availability

Data sharing not applicable to this article as no datasets were generated or analyzed during the current study.

## References

[1] S. A. Vargas , G. R. T. Esteves , P. M. Maçaira , B. Q. Bastos , F. L. C. Oliveira , and R. C. Souza , “Wind Power Generation: A Review and a Research Agenda,” Journal of Cleaner Production 218 (2019): 850.

[2] M. Z. Jacobson , M. A. Delucchi , Z. A. F. Bauer , et al., “100% Clean and Renewable Wind, Water, and Sunlight All‐Sector Energy Roadmaps for 139 Countries of the World,” Joule 1 (2017): 108.

[3] R. A. Kerr , “Global Warming Is Changing the World,” Science 316 (2007): 188. 17431148 10.1126/science.316.5822.188

[4] Z. Zhu , T. Jiang , M. Ali , et al., “Rechargeable Batteries for Grid Scale Energy Storage,” Chemical Reviews 122 (2022): 16610. 36150378 10.1021/acs.chemrev.2c00289

[5] T. Jiang , D. Shen , Z. Zhang , et al., “Battery Technologies for Grid‐Scale Energy Storage,” Nature Reviews Clean Technology 1 (2025): 474.

[6] J.‐H. Kim , N.‐Y. Kim , Z. Ju , et al., “Upscaling High‐Areal‐Capacity Battery Electrodes,” Nature Energy 10 (2025): 295.

[7] J. R. Owen , D. Kemp , A. M. Lechner , J. Harris , R. L. Zhang , and É. Lèbre , “Energy Transition Minerals and Their Intersection with Land‐Connected Peoples,” Nature Sustainability 6 (2023): 203.

[8] Z. Hao , X. Shi , Z. Yang , et al., “The Distance Between Phosphate‐Based Polyanionic Compounds and Their Practical Application for Sodium‐Ion Batteries,” Advanced Materials 36, 2023): 2305135. 10.1002/adma.202305135 37590909

[9] L. Yang , X. Yin , J. Wang , et al., “Substitution and Electrochemistry in Layered Oxide Cathode Materials for Sodium‐Ion Batteries,” Nature Reviews Chemistry 10 (2026): 1–6. 10.1038/s41570-025-00795-3 41611985

[10] B. Song , Y. Cheng , G. Zhao , et al., “Sodium Ion Batteries: From Basic Research to Industrialization,” Advanced Functional Materials 35 (2025): e10872.

[11] S. Mariyappan , P. Desai , M. Morcrette , and J.‐M. Tarascon , “From Lab to Market with Sustainable Sodium‐Ion Batteries,” Nature Sustainability 9 (2025): 360–371.

[12] C. Liu , Z. Zhang , H. Y. Liao , et al., “Piezophototronic Effect‐Enhanced Highly Sensitive Flexible Photodetectors Based on Electrohydrodynamic Direct‐Writing Nanofiber Self‐Stacking,” Advanced Functional Materials 7 (2025): 35.

[13] Q. N. Zhou , Y. Li , H. X. Ren , et al., “Phase Transition and Targeted Modulation Mechanisms of Layered Cathodes for Sodium‐Ion Batteries,” Materials Today 89 (2025): 374.

[14] X. Gao , L. Guo , S. Zhang , et al., “Unveiling the Influence of Cyanogen Vacancies in Prussian Blue for Sodium‐ion Batteries,” Angewandte Chemie International Edition 64 (2025): e202421916. 40574644 10.1002/anie.202421916

[15] W. D. Song , N. Chen , J. P. Wang , et al., “Mechanistic Insights into Enhanced Capacity and Pure‐Phase Formation in Fe‐Based Mixed Phosphate Cathodes,” Journal of the American Chemical Society 147 (2025): 39151. 41100871 10.1021/jacs.5c08636

[16] C. Sun , J. Chen , H. Hou , et al., “Covalency Regulation of Metal‐Oxygen Ligand in O3‐Type Layered Cathode Material for High‐Performance Sodium‐Ion Batteries,” Energy Storage Materials 84 (2026): 104820.

[17] Z. Q. Yang , J. S. Xie , Y. Li , et al., “Tailored Interphase Chemistry Enables Ultra‐Stable O3‐Type Sodium Layered Oxide Cathodes,” Journal of the American Chemical Society 147 (2025): 44060. 41230733 10.1021/jacs.5c10193

[18] Y. Wang , J. Yan , B. Xie , et al., “Tuning Cyanide Coordination Electronic Structure Enables Stable Prussian Blue Analogues for Sodium‐Ion Batteries,” Nature Communications 16 (2025): 10083. 10.1038/s41467-025-65062-x PMC12627476 41253817

[19] X. Lin , B. Zhou , S. Xu , et al., “Hydrophobic Lattice Engineering of Prussian Blue Analogs with Accelerated Redox Kinetics for High‐Areal‐Capacity Sodium‐Ion Battery Electrodes,” ACS Nano 19 (2025): 31023. 40824754 10.1021/acsnano.5c08791

[20] X. Chen , K. Chen , F. Ji , et al., “Achieving Fast Ion/Electron Transportation and Smooth Phase Transition in Polyanion Cathode by the High‐Entropy Strategy,” Advanced Energy Materials 15 (2025): 2500502.

[21] H. Zhang , X. T. Wang , W. Y. Qian , et al., “Electronic Cloud Topology‐Driven Electrostatic Decoupling: To Suppress High‐Voltage Parasitic Reactions of Phosphate Cathode in Sodium‐Ion Batteries,” Angewandte Chemie International Edition 64 (2025): 64. 10.1002/anie.202510387 40781821

[22] C. Jin , Y. Wang , X. Zhao , et al., “Entropy Driving “Quasi‐Zero Strain” Stepwise Multicationic Redox Chemistry Toward a High‐Performance NASICON‐Cathode for Na‐Ion Batteries,” Advanced Functional Materials 35 (2025): 2422101.

[23] Y. L. Yan , W. X. Zhao , J. Q. Hu , et al., Advanced Energy Materials (2026).

[24] C. Xu , J. Zhao , C. Yang , and Y.‐S. Hu , “Polyanionic Cathode Materials for Practical Na‐Ion Batteries toward High Energy Density and Long Cycle Life,” ACS Central Science 9 (2023): 1721. 37780368 10.1021/acscentsci.3c00907 PMC10540287

[25] A. Zhao , T. Yuan , P. Li , et al., “A Novel Fe‐Defect Induced Pure‐Phase Na_4_Fe_2.91_(PO_4_)_2_P_2_O_7_ Cathode Material with High Capacity and Ultra‐Long Lifetime for Low‐Cost Sodium‐Ion Batteries,” Nano Energy 91 (2022): 106680.

[26] W. Jian , L. Sun , J. Gao , et al., “Valence‐Modulated Na_4_Fe_3_(PO_4_)_2_(P_2_O_7_) Cathode Tuned by Orbital‐Delocalization for Extreme‐Temperature Sodium Storage,” Angewandte Chemie International Edition 64 (2025): e202514523. 41074821 10.1002/anie.202514523

[27] H. Xia , W. Xu , W. Wang , et al., “Na_4_Fe_3_(PO_4_)_2_P_2_O_7_ Cathode for Sodium‐Ion Batteries: Critical Technologies and Progress from Fundamental Advances to Industrialization Challenges,” Energy Storage Materials 84 (2026): 104788.

[28] C. Liu , Z. Zhang , H. Liao , et al., “Unlocking the Potential: Na_4_Fe_3_(PO_4_)_2_(P_2_O_7_) Supporting the Innovation of Commercial Sodium‐Ion Batteries,” Advanced Functional Materials 35 (2025): 2424759.

[29] L. Ran , B. Shen , L. Yue , et al., “Accelerating Electrochemical Kinetics in Na_4_Fe_3_(PO_4_)_2_P_2_O_7_ Cathodes Through Oxygen Vacancy Modulation for Wide‐Temperature Ah‐Level Sodium‐Ion Batteries,” Angewandte Chemie International Edition (2026): e25531, 10.1002/anie.202525531. 41549674

[30] H. Kim , I. Park , D.‐H. Seo , et al., “New Iron‐Based Mixed‐Polyanion Cathodes for Lithium and Sodium Rechargeable Batteries: Combined First Principles Calculations and Experimental Study,” Journal of the American Chemical Society 134 (2012): 10369. 22667817 10.1021/ja3038646

[31] X. Wu , G. Zhong , and Y. Yang , “Sol‐Gel Synthesis of Na_4_Fe_3_(PO_4_)_2_(P_2_O_7_)/C Nanocomposite for Sodium Ion Batteries and New Insights into Microstructural Evolution during Sodium Extraction,” Journal of Power Sources 327 (2016): 666.

[32] H. Kim , I. Park , S. Lee , et al., “Understanding the Electrochemical Mechanism of the New Iron‐Based Mixed‐Phosphate Na_4_Fe_3_(PO_4_)_2_(P_2_O_7_) in a Na Rechargeable Battery,” Chemistry of Materials 25 (2013): 3614.

[33] Y. Wang , F. X. Deng , S. W. Ouyang , C. Jiang , and H. X. Li , “Current Progress of Na4Fe3(PO4)2(P2O7): Key Issues, Modifications, and Perspectives,” Journal of Energy Chemistry 111 (2025): 914.

[34] X. Y. Shi , Z. Q. Hao , W. Q. Zhu , et al., “Na4Fe3(PO4)2(P2O7)/C Composite with Porous Structure Enabling All‐Climate and Long‐Life Sodium‐Ion Batteries,” Science China Materials 67 (2024): 3622.

[35] X. R. Qi , Q. Y. Dong , H. H. Dong , et al., ENERGY STORAGE MATERIALS. (2024): 73.

[36] M. Chen , W. Hua , J. Xiao , et al., “NASICON‐Type Air‐Stable and All‐Climate Cathode for Sodium‐Ion Batteries with Low Cost and High‐Power Density,” Nature Communications 10 (2019): 1480. 10.1038/s41467-019-09170-5 PMC6443767 30931938

[37] X. Xiao , Y. Lan , L. Tan , H. Xu , W. Yao , and Y. Tang , “Alluaudite Na2Fe2(SO4)3 and NASICON‐Type Na4Fe3(PO4)2(P2O7) as Promising Cathode Materials in Sodium‐Ion Batteries,” Advanced Functional Materials 34 (2024): 2411280.

[38] S. M. Wood , C. Eames , E. Kendrick , and M. S. Islam , “Sodium Ion Diffusion and Voltage Trends in Phosphates Na_4_M_3_(PO_4_)_2_P_2_O_7_ (M = Fe, Mn, Co, Ni) for Possible High‐Rate Cathodes,” Journal of Physical Chemistry C 119 (2015): 15935.

[39] S. Baskar , R. Angalakuthi , C. Murugesan , S. B. Krupanidhi , and P. Barpanda , “Exploration of Iron‐Based Mixed Polyanion Cathode Material for Thin‐Film Sodium‐Ion Batteries,” ECS Transactions 85 (2018): 227.

[40] H. Kim , R. A. Shakoor , C. Park , et al., “Na_2_FeP_2_O_7_ as a Promising Iron‐Based Pyrophosphate Cathode for Sodium Rechargeable Batteries: A Combined Experimental and Theoretical Study,” Advanced Functional Materials 23 (2012): 1147.

[41] P. Moreau , D. Guyomard , J. Gaubicher , and F. Boucher , “Structure and Stability of Sodium Intercalated Phases in Olivine FePO_4_ ,” Chemistry of Materials 22 (2010): 4126.

[42] X. Li , S. Dai , Q. Chen , H. Mao , and H. Pan , “Understanding the Air Sensitivity and Deterioration Mechanism of the Na_4_Fe_3_(PO_4_)_2_P_2_O_7_ Cathode for Na‐Ion Batteries,” Journal of Materials Chemistry A 12 (2024): 29726.

[43] Q. Tao , H. Ding , X. Tang , et al., “Mn‐Doped Na_4_Fe_3_(PO_4_)_2_P_2_O_7_ as a Low‐Cost and High‐Performance Cathode Material for Sodium‐Ion Batteries,” Energy & Fuels 37 (2023): 6230.

[44] T. Chen , X. Han , M. Jie , Z. Guo , J. Li , and X. He , “Mo‐Doped Na_4_Fe_3_(PO_4_)_2_P_2_O_7_/C Composites for High‐Rate and Long‐Life Sodium‐Ion Batteries,” Materials 17 (2024): 2679. 38893941 10.3390/ma17112679 PMC11174099

[45] W. Ren , M. Qin , Y. Zhou , et al., “Electrospun Na4Fe3(PO4)2(P2O7) Nanofibers as Free‐Standing Cathodes for Ultralong‐Life and High‐Rate Sodium‐Ion Batteries,” Energy Storage Materials 54 (2023): 776.

[46] X. Pu , H. Wang , T. Yuan , et al., “Na_4_Fe_3_(PO_4_)_2_P_2_O_7_/C Nanospheres as Low‐Cost, High‐Performance Cathode Material for Sodium‐Ion Batteries,” Energy Storage Materials 22 (2019): 330.

[47] J. Gao , H. Chen , Y. Mei , et al., ”Robust Iron‐Based Cathode for Ultralong‐Lasting Na‐Ion Battery with a Wide Operation Temperature,” Nano Energy 115 (2023): 108747.

[48] T. Yuan , Y. Wang , J. Zhang , et al., “3D Graphene Decorated Na4Fe3(PO4)2(P2O7) Microspheres as Low‐Cost and High‐Performance Cathode Materials for Sodium‐Ion Batteries,” Nano Energy 56 (2019): 160.

[49] C. Xu , L. Zhou , T. Gao , et al., “Development of High‐Performance Iron‐Based Phosphate Cathodes toward Practical Na‐Ion Batteries,” Journal of the American Chemical Society 146 (2024): 9819. 38546207 10.1021/jacs.3c14452

[50] N. V. Kosova and V. A. Belotserkovsky , “Sodium and Mixed Sodium/Lithium Iron Ortho‐Pyrophosphates: Synthesis, Structure and Electrochemical Properties,” Electrochimica Acta 278 (2018): 182.

[51] X. Ge , B. Zhu , L. He , X. Wang , Y. Lai , and Z. Zhang , ”Toward High Performance of Na_4_Fe_3_(PO_4_)_2_P_2_O_7_ Cathode via Constructing a Porous Structure for Sodium‐Ion Batteries,” ACS Sustainable Chemistry & Engineering 12, 2024): 11361–11368.

[52] H. Wang , Z. Pan , H. Zhang , et al., “A Green and Scalable Synthesis of Na_3_Fe_2_(PO_4_)P_2_O_7_/rGO Cathode for High‐Rate and Long‐Life Sodium‐Ion Batteries,” Small Methods 5 (2021): 2100372. 10.1002/smtd.202100372 34927871

[53] Y. Chen , C. Dong , L. Chen , et al., “One Stone, Two Birds” Design for Hollow Spherical Na_4_Fe_3_(PO_4_)_2_P_2_O_7_/C Cathode Enabled High‐Performance Sodium‐Ion Batteries from Iron Rust,” Ecomat 5 (2023): e12393.

[54] L.‐m. Zhang , X.‐d. He , S. Wang , et al., “Hollow‐Sphere‐Structured Na_4_Fe_3_(PO_4_)_2_(P_2_O_7_)/C as a Cathode Material for Sodium‐Ion Batteries,” ACS Applied Materials and Interfaces 13 (2021): 25972. 34038077 10.1021/acsami.1c04035

[55] B. Senthilkumar , C. Murugesan ,8 K. Sada , and P. Barpanda , “Electrochemical Insertion of Potassium Ions in Na_4_Fe_3_(PO_4_)_2_P_2_O_7_ Mixed Phosphate,” Journal of Power Sources 480 (2020): 228794.

[56] P. Barpanda , G. Liu , C. D. Ling , et al., “Na_2_FeP_2_O_7_: A Safe Cathode for Rechargeable Sodium‐Ion Batteries,” Chemistry of Materials 25 (2013): 3480.

[57] A. Gezović , M. Milović , D. Bajuk‐Bogdanović , et al., “An Effective Approach to Reaching the Theoretical Capacity of a Low‐Cost and Environmentally Friendly Na_4_Fe_3_(PO_4_)_2_(P_2_O_7_) Cathode for Na‐Ion Batteries,” Electrochimica Acta 476 (2024): 143718.

[58] J. Wu , L. Xiao , P. Liu , Y. Zhu , and J. Li , “3D Interconnected Porous Sodium Super Ionic Conductor‐Structured Na_4_Fe_3_(PO_4_)_2_(P_2_O_7_)/C as a Cathode Material for Sodium‐Ion Batteries,” Energy and Fuels 38 (2024): 19117.

[59] J. Zhang , L. Tang , Y. Zhang , et al., “Polyvinylpyrrolidone Assisted Synthesized Ultra‐Small Na_4_Fe_3_(PO_4_)_2_(P_2_O_7_) Particles Embedded in 1D Carbon Nanoribbons with Enhanced Room and Low Temperature Sodium Storage Performance,” Journal of Power Sources 498 (2021): 229907.

[60] A. Zhao , C. Liu , F. Ji , et al., “Revealing the Phase Evolution in Na_4_Fe_ *x* _P_4_O_12+*x* _(2 ≤ *x* ≤ 4) Cathode Materials,” ACS Energy Letters 8 (2022): 753.

[61] Z. Fan , W. Song , N. Yang , et al., “Insights into the Phase Purity and Storage Mechanism of Nonstoichiometric Na_3.4_Fe_2.4_(PO_4_)_1.4_P_2_O_7_ Cathode for High‐Mass‐Loading and High‐Power‐Density Sodium‐Ion Batteries,” Angewandte Chemie International Edition 63 (2024): e202316957. 38168896 10.1002/anie.202316957

[62] Y. Wang , W. Fei , X. Zhang , et al., “Rapid Mechanochemical Synthesis of High‐Performance Na_4_Fe_2.94_Al_0.04_(PO_4_)_2_(P_2_O_7_)/C Cathode Material for Sodium‐Ion Storage,” Journal of Colloid and Interface Science 664 (2024): 220. 38461788 10.1016/j.jcis.2024.03.036

[63] R. Liu , Z. Liang , Z. Gong , and Y. Yang , “Research Progress in Multielectron Reactions in Polyanionic Materials for Sodium‐Ion Batteries,” Small Methods 3 (2019): 1800221.

[64] H. Ding , X. Li , H. Li , and J. He , “A Nonstoichiometric Pure‐Phase Na_3.4_Fe_2.4_(PO_4_)_1.4_P_2_O_7_ Cathode for High‐Performance Sodium‐Ion Batteries,” ACS Sustainable Chemistry & Engineering 12 (2024): 10528.

[65] H. Yang , X. Li , Z. Wang , et al., “Phase Regulation Promotes High Rate‐Long Term Na_4_Fe_3_(PO_4_)_2_P_2_O_7_ Cathode for Sodium‐Ion Batteries,” Batteries & Supercaps 7 (2024): e202400438.

[66] H. Zhang , Y. Cao , Z. Liu , et al., “Structurally Modulated Na_4–*x* _Fe_3–*x* _V_ *x* _(PO_4_)_2_P_2_O_7_ by Vanadium Doping for Long‐Life Sodium‐Ion Batteries,” ACS Sustainable Chemistry & Engineering 12 (2024): 5310.

[67] W. Fei , Y. Wang , X. Zhang , et al., “A Novel Bimetallic‐Polyanion Na_4_Fe_2.82_Ni_0.18_(PO_4_)_2_P_2_O_7_ Cathode with Superior Rate Performance and Long Cycle‐Life for Sodium‐Ion Batteries,” Chemical Engineering Journal 493 (2024): 152523.

[68] W. Fei , X. Zhang , K. Sun , et al., “Dual‐Site Defects Engineering to Eliminate Impurities and Optimize Reversible Reaction Kinetics of Na_4_Fe_3_(PO_4_)_2_P_2_O_7_ Cathode for Superior Performance Sodium Ion Batteries,” Energy Storage Materials 73 (2024): 103848.

[69] R. Z. Ma , J. Q. Meng , X. P. Su , et al., “A Bifunctional Na‐Deficient Strategy Induced Pure Phase Na_4−*x* _Fe_3_(PO_4_)_2_P_2_O_7_ Cathode with High Capacity for Sodium‐Ion Batteries,” Journal of Materials Chemistry A 13 (2025): 2631.

[70] G.‐L. Xu , R. Amine , A. Abouimrane , et al., “Challenges in Developing Electrodes, Electrolytes, and Diagnostics Tools to Understand and Advance Sodium‐Ion Batteries,” Advanced Energy Materials 8 (2018): 1702403.

[71] X. Xiang , K. Zhang , and J. Chen , “Recent Advances and Prospects of Cathode Materials for Sodium‐Ion Batteries,” Advanced Materials 27 (2015): 5343. 26275211 10.1002/adma.201501527

[72] F. Xiong , H. Tao , and Y. Yue , “Role of Amorphous Phases in Enhancing Performances of Electrode Materials for Alkali Ion Batteries,” Frontiers in Materials 6 (2020).

[73] J. Z. Guo , H. X. Zhang , Z. Y. Gu , et al., “Heterogeneous NASICON‐Type Composite as Low‐Cost, High‐Performance Cathode for Sodium‐Ion Batteries,” Advanced Functional Materials 32 (2022): 2209482.

[74] X. Ma , Z. Pan , X. Wu , and P. K. Shen , “Na_4_Fe_3_(PO_4_)_2_(P_2_O_7_)@NaFePO_4_@C Core‐Double‐Shell Architectures on Carbon Cloth: A High‐Rate, Ultrastable, and Flexible Cathode for Sodium Ion Batteries,” Chemical Engineering Journal 365 (2019): 132.

[75] L. Zhu , S. Xiang , M. Wang , et al., “Free‐Boundary Microfluidic Platform for Advanced Materials Manufacturing and Applications,” Advanced Materials 36 (2024): 2408918. 10.1002/adma.202304840 37722080

[76] H. Dai , Y. Xu , Y. Wang , et al., “Entropy‐Driven Enhancement of the Conductivity and Phase Purity of Na_4_Fe_3_(PO_4_)_2_P_2_O_7_ as the Superior Cathode in Sodium‐Ion Batteries,” ACS Applied Materials & Interfaces 16 (2024): 7070. 38308393 10.1021/acsami.3c15947

[77] H. Wu , T. Wen , L. Chen , et al., “Understanding the Role of Mn Substitution for Boosting High‐Voltage Na_4_Fe_3−*x* _Mnx(PO_4_)_2_P_2_O_7_ Cathode in Sodium‐Ion Batteries,” Small Methods 9, no. 1 (2024): 2400642, 10.1002/smtd.202400642. 39155809

[78] X. Li , Y. Zhang , B. Zhang , K. Qin , H. Liu , and Z.‐F. Ma , “Mn‐Doped Na_4_Fe_3_(PO_4_)_2_(P_2_O_7_) Facilitating Na+ Migration at Low Temperature as a High Performance Cathode Material of Sodium Ion Batteries,” Journal of Power Sources 521 (2022): 230922.

[79] X. Qi , H. Dong , H. Yan , et al., “Hollow Core‐Shelled Na_4_Fe_2.4_Ni_0.6_(PO_4_)_2_P_2_O_7_ with Tiny‐Void Space Capable Fast‐Charge and Low‐Temperature Sodium Storage,” Angewandte Chemie‐International Edition 136 (2024): e202410590. 10.1002/anie.202410590 38888029

[80] J. Gao , J. Zeng , W. Jian , et al., “Aluminum Ion Chemistry of Na_4_Fe_3_(PO_4_)_2_(P_2_O_7_) for All‐Climate Full Na‐Ion Battery,” Science Bulletin 69 (2024): 772. 38310048 10.1016/j.scib.2024.01.026

[81] Y. Han , X. Wang , W. Yan , et al., “Solid‐State Synthesis of Na_4_Fe_3_(PO_4_)_2_P_2_O_7_/C by Ti‐Doping with Promoted Structural Reversibility for Long‐Cycling Sodium‐Ion Batteries,” ACS Applied Materials & Interfaces 16 (2024): 35114. 38941158 10.1021/acsami.4c05943

[82] L. Huang , C. Liu , L. Bao , Y. Chen , Y. Jiang , and X. Fu , “Large Scalable Preparation of Ti‐Doped Na_4_Fe_3_(PO_4_)_2_P_2_O_7_ as Cathode Material for High Rate and Long‐Life Sodium‐Ion Batteries,” ACS Applied Energy Materials 6 (2023): 11541.

[83] X. Wang , H. X. Li , W. Zhang , et al., “Unlocking Fast and Highly Reversible Sodium Storage in Fe‐Based Mixed Polyanion Cathodes for Low‐Cost and High‐Performance Sodium‐Ion Batteries,” Journal of Materials Chemistry A 11 (2023): 6978.

[84] S. Xu , J. Yuan , D. Ma , et al., “Hollow Spherical Na_3.95_Fe_2.95_V_0.05_(PO_4_)_2_P_2_O_7_ Suppressing Inactive Maricite‐NaFePO4 with Ultrahigh Dynamics Performance,” Nano Energy 132 (2024): 110404.

[85] N. Jiang , C. Yang , Y. Wang , X. Wang , S. Sun , and Y. Liu , “Vacancy and Low‐Energy 3p‐Orbital Endow Na_4_Fe_3_(PO_4_)_2_(P_2_O_7_) Cathode with Superior Sodium Storage Kinetics,” Small. (2025): e2410715. 39757500 10.1002/smll.202410715

[86] F. Peng , P. Dong , C. Chen , et al., “Spherical Mg/Cu Co‐Doped Na_4_Fe_3_(PO_4_)_2_P_2_O_7_ Cathode Materials with Mitigated Diffusion‐Induced Stresses and Enhanced Cyclic Stability,” Angewandte Chemie International Edition 64 (2025): e202423296. 39714588 10.1002/anie.202423296

[87] W. S. Jian , L. Sun , J. Q. Gao , et al., Angewandte Chemie‐international Edition. (2025): 64.

[88] X. Qi , Q. Dong , H. Dong , et al., “Copper‐Induced Lattice Distortion in Na_4_Fe_3_(PO_4_)_2_(P_2_O_7_) Cathode Enabling High Power Density Na‐Ion Batteries with Good Cycling Stability,” Energy Storage Materials 73 (2024): 103861.

[89] M. Liu , M. Li , B. Zhang , et al., “Anionic Group Doping of Na_4_Fe_3_(PO_4_)_2_P_2_O_7_ Stabilizes Its Structure and Improves Electrochemical Performance for Sodium Ion Storage,” ACS Sustainable Chemistry & Engineering 11 (2023): 18102.

[90] N. Jiang , X. Wang , H. Zhou , et al., “Achieving Fast and Stable Sodium Storage in Na_4_Fe_3_(PO_4_)_2_(P_2_O_7_) via Entropy Engineering,” Small 20 (2024): 2308681. 10.1002/smll.202308681 38234151

[91] Y. Xin , Q. Wang , Y. Wang , M. Wang , F. Wu , and H. Gao , “Experimental and Theoretical Investigation of Cobalt and Manganese Substitution in Na_4_Fe_3_(PO_4_)_2_P_2_O_7_ as a High Energy Density Cathode Material for Sodium‐Ion Batteries,” Chemical Engineering Journal 483 (2024): 149438.

[92] Y. Xi , X. Wang , H. Wang , et al., “Optimizing the Electron Spin States of Na_4_Fe_3_(PO_4_)_2_P_2_O_7_ Cathodes via Mn/F Dual‐Doping for Enhanced Sodium Storage,” Advanced Functional Materials. 34 (2024): 2309701.

[93] X. Ge , L. He , C. Guan , et al., “Anion Substitution Strategy toward an Advanced NASICON‐Na_4_Fe_3_(PO_4_)_2_P_2_O_7_ Cathode for Sodium‐Ion Batteries,” ACS Nano 18 (2024): 1714. 38156873 10.1021/acsnano.3c10319

[94] Q. Lu , T. Y. Shen , C. L. Li , et al., “Bond‐Defect Synergy Enabled Ultrastable and High‐Rate Sodium Iron Phosphate Cathode through Zn/F Co Substitution,” Nano Letters 26 (2026): 1375. 41554633 10.1021/acs.nanolett.5c05384

[95] G. Li , Y. Cao , J. Chen , et al., “Entropy‐Enhanced Multi‐Doping Strategy to Promote the Electrochemical Performance of Na_4_Fe_3_(PO_4_)_2_P_2_O_7_ ,” Small Methods 8 (2024): 2301745. 10.1002/smtd.202301745 38326032

[96] H. Wang , Z. Zhang , Y. Chu , et al., “Boosting Na ^+^ Storage and Thermal Stability of Na_4_Fe_3_(PO_4_)_2_P_2_O_7_ via High‐Entropy Engineering,” ACS Nano 19 (2025): 41824. 41346278 10.1021/acsnano.5c15785

[97] Y. Yan , W. Zhao , J. Hu , et al., Advanced Energy Materials n/a (2026): e05808.

[98] X. Ge , H. Li , J. Li , et al., ”High‐Entropy Doping Boosts Ion/Electronic Transport of Na_4_Fe_3_(PO_4_)_2_(P_2_O_7_)/C Cathode for Superior Performance Sodium‐Ion Batteries,” Small 19 (2023): 2302609. 10.1002/smll.202302609 37140083

[99] Y. Qiu , Q. Shi , X. Yu , et al., “High‐Entropy Na_4_Fe_2.65_(NiCrMgCoMn)_0.027_(PO_4_)_2_P_2_O_7_ Cathode for High‐Rate Sodium‐Ion Batteries,” Chemical Engineering Science 300 (2024): 120671.

[100] X. Hu , S. Liang , J. Lin , et al., “Synergistic Configurational Entropy and Iron Vacancy Engineering in Na4Fe3 (PO4) 2P2O7 Cathode for High‐Power‐Density and Ultralong‐Life Na‐Ion Full Batteries,” Advanced Energy Materials 15, no. 17 (2024): 2404965, 10.1002/aenm.202404965.

[101] J. Gao , Y. Tian , Y. Mei , et al., “Robust NASICON‐Type Iron‐Based Na_4_Fe_3_(PO_4_)_2_(P_2_O_7_) Cathode for High Temperature Sodium‐Ion Batteries,” Chemical Engineering Journal 458 (2023): 141385.

[102] X. Li , J. Zhang , Y. Zhang , et al., “A Facile Ball‐Milling Preparation Strategy of Nitrogen‐Doped Carbon Coated Na_4_Fe_3_(PO_4_)_2_P_2_O_7_ Nano‐Flakes with Superior Sodium Ion Storage Performance,” Chemical Engineering Science 260 (2022): 117951.

[103] J. Huang , Z. Zhang , D. Chen , H. Yu , Y. Wu , and Y. Chen , “Spray‐Drying Synthesis of Na_4_Fe_3_(PO_4_)_2_P_2_O_7_@CNT Cathode for Ultra‐Stable and High‐Rate Sodium‐Ion Batteries,” Molecules 30 (2025): 753. 39942856 10.3390/molecules30030753 PMC11819995

[104] Y. Cao , X. Xia , Y. Liu , et al., “Scalable Synthesizing Nanospherical Na_4_Fe_3_(PO_4_)_2_(P_2_O_7_) Growing on MCNTs as a High‐Performance Cathode Material for Sodium‐Ion Batteries,” Journal of Power Sources 461 (2020): 228130.

[105] Z. Li , F. Li , X. Xu , et al., “A Scalable Approach to Na_4_Fe_3_(PO_4_)_2_P_2_O_7_@Carbon/Expanded Graphite as Cathode for Ultralong‐Lifespan and Low‐Temperature Sodium‐Ion Batteries,” Chinese Chemical Letters 36 (2025): 110390, 10.1016/j.cclet.2024.110390.

[106] A. Xiang , D. Shi , P. Chen , et al., ”Na_4_Fe_3_(PO_4_)_2_(P_2_O_7_)@C/Ti_3_C_2_T_x_ Hybrid Cathode Materials with Enhanced Performances for Sodium‐Ion Batteries,” Batteries, 10 (2024): 121.

[107] X. Li , Y. Meng , and D. Xiao , “Three‐Dimensional Holey Graphene Modified Na_4_Fe_3_(PO_4_)_2_(P_2_O_7_)/C as a High‐Performance Cathode for Rechargeable Sodium‐Ion Batteries,” Chemistry‐a European Journal 29 (2023): e202203381. 36448358 10.1002/chem.202203381

[108] X. Ma , X. Wu , and P. Shen , “Rational Design of Na_4_Fe_3_(PO_4_)_2_(P_2_O_7_) Nanoparticles Embedded in Graphene: Toward Fast Sodium Storage Through the Pseudocapacitive Effect,” ACS Applied Energy Materials 1 (2018): 6268.

[109] Z. Li , Y. Zhang , J. Zhang , et al., “Sodium‐Ion Battery with a Wide Operation‐Temperature Range from −70 to 100 °C,” Angewandte Chemie International Edition 61 (2022): e202116930. 35044037 10.1002/anie.202116930

[110] Y. Cao , G. Li , J. Chen , et al., “A Presodiation Strategy to Extend the Cycle Life of Iron Phosphate Sodium‐Ion Full Cell,” Journal of Power Sources 587 (2023): 233718.

[111] H. Chen , K. Chen , J. Yang , et al., “Designing Advanced Electrolytes for High‐Safety and Long‐Lifetime Sodium‐Ion Batteries via Anion–Cation Interaction Modulation,” Journal of the American Chemical Society 146 (2024): 15751. 38833380 10.1021/jacs.4c01395

[112] J. Y. Jang , H. Kim , Y. Lee , K. T. Lee , K. Kang , and N.‐S. Choi , “Cyclic Carbonate Based‐Electrolytes Enhancing the Electrochemical Performance of Na_4_Fe_3_(PO_4_)_2_(P_2_O_7_) Cathodes for Sodium‐Ion Batteries,” Electrochemistry Communications 44 (2014): 74.

